# Constructing Palladium‐Based Crystalline@Amorphous Core–Shell Heterojunctions for Efficient Formic Acid Oxidation

**DOI:** 10.1002/advs.202504469

**Published:** 2025-04-30

**Authors:** Huiling Li, Jingkun Yu, Yongming Sui, Weibin Wang, Jiewen Liu, LiBo Sheng, Ankang Chen, Siyu Lu, Bo Zou

**Affiliations:** ^1^ State Key Laboratory of High Pressure and Superhard Materials College of Physics Jilin University China 2699 Qianjin Street Changchun 130012 China; ^2^ College of Chemistry and Pingyuan Laboratory Zhengzhou University Zhengzhou 45000 China

**Keywords:** core–shell structure, crystalline@amorphous, formic acid oxidation, heterojunctions, palladium‐based catalyst

## Abstract

Constructing crystalline@amorphous heterostructures allows nanomaterials to maintain high electrical conductivity of crystalline structures while acquiring abundant active sites from amorphous structure. This emerging strategy has attracted considerable attention in electrochemical and photoelectrochemistry applications. However, achieving crystalline@amorphous heterostructures based on palladium (Pd) remains challenging due to the difficulties in balancing the transformation between these two phases. Here, a feasible strategy is developed to manufacture Pd‐based crystalline@amorphous core–shell structures through non‐metallic element doping. The obtained core–shell structures exhibit outstanding catalytic performance for formic acid oxidation (FAO) with mass activity of up to 2.503 A mg^−1^_Pd_. Detailed theoretical and experimental analyses reveal that the construction of crystalline@amorphous core–shell structures increase surface active sites, lowers the oxidation energy barrier, and enhances the selectivity of the direct pathway, thereby effectively facilitating the FAO process. This work demonstrates the feasibility of constructing efficient FAO catalysts using crystalline@amorphous core–shell structures and provides a new platform for achieving platinum‐group metals (PGMs) based crystalline‐amorphous heterostructures.

## Graphical abstract

Crystalline@amorphous core–shell PdCN‐P nanocubes exhibit outstanding catalytic performance for formic acid oxidation, with mass activity and specific activity reaching 2.503 A mg^−1^_Pd_ and 6.497 mA cm^−2^, respectively. Detailed theoretical and experimental analyses indicate that the unique core‐shell architecture can increase surface active sites, lower the oxidation energy barrier, and enhance the selectivity of the direct pathway, thereby facilitating FAO process.

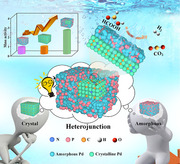

## 1 Introduction

Direct formic acid fuel cells (DFAFCs), known for their considerable energy density, low operating temperature and easy management, are among the most promising power sources for future portable electronic devices.^[^ ^1^ ^]^ The primary challenge in commercializing DFAFCs lies in the development of catalysts with high‐performance, and cost‐effective for formic acid oxidation (FAO).^[^ ^2^ ^]^ In this regard, platinum‐group metals (PGMs) based crystalline materials are generally considered the most efficient catalysts for FAO.^[^ ^1^, ^3^ ^]^ During the past decades, the activity of these PGMs‐based catalysts have been significantly improved by employing advanced strategies, including alloying, doping and morphology tailoring.^[^ ^4^ ^]^ However, the catalytic stability of PGMs‐based crystalline materials is still limited due to lack of the structural flexibility and corrosion resistance.^[^ ^5^ ^]^ Developing new arsenal of catalysts, which address the requirements for practical applications of DFAFCs, is desirable but remains a formidable challenge.

Recently, crystalline‐amorphous heterojunctions, which combines the high conductivity of crystalline phase and the ample active sites of amorphous phase, have attracted considerable attention in electrochemistry and photo‐electrochemistry.^[^ ^5^, ^6^ ^]^ The pursuit of crystalline‐amorphous heterojunction based on PGMs not only opens new avenues in phase engineering of the nanomaterials, but also offers an alternative to traditional crystalline catalysts in FAO.^[^ ^7^ ^]^ For instance, Zhang et al. obtained crystalline‐amorphous Pd nanosheets through a one‐pot wet‐chemical method.^[^ ^8^ ^]^ Subsequent studies revealed that the synergistic effect of crystallization and amorphous is beneficial for the activation of C‐H and O‐H bonds, which significantly improves the specific activity of these nanosheets in FAO.^[^ ^7a^ ^]^ Despite these achievements, fundamental questions remain over the structure‐property relationship of the crystalline‐amorphous heterojunctions in PGMs based catalysts. Some studies have shown that heteroatom ensemble (HAE) effect can be created in these heterojunctions, which increasing the electrochemical surface area (ECSA) of the catalyst.^[^ ^9^ ^]^ Nevertheless, the specific ensemble required by FAO reaction remains elusive in crystalline‐amorphous heterojunctions based on PGMs. In addition, crystalline‐amorphous heterojunctions with core–shell structures typically demonstrate enhanced electrochemical stability.^[^ ^5b^ ^]^ Still, only a few studies have successfully developed well‐defined core–shell crystalline‐amorphous nanomaterials based on PGMs due to the fast amorphization kinetics.^[^ ^6e^ ^]^

Herein, we developed non‐metallic element doping as an feasible method for constructing crystalline@amorphous core–shell nanocubes (NCs) with tunable shell thickness. Our approach utilizes palladium (Pd) NCs as a foundational structure, with carbon (C) and nitrogen (N) incorporated to retard their amorphization kinetics, resulting in PdCN NCs. Subsequently, phosphorus (P) was introduced through substitutional doping to induce surface amorphization of the PdCN nanocubes.^[^ ^10^ ^]^ The obtained crystalline@amorphous core–shell NCs (referred to as PdCN‐P NCs) exhibited tunable shell thickness and shell‐thickness‐dependent FAO catalytic performance. PdCN‐P NCs with a 2.8 nm amorphous shell demonstrated mass activity of up to 2.503 A mg^−1^_Pd,_ which is 17.88 and 8.26 times higher than that of Pd black and Pd NCs, respectively. Detailed experimental and theoretical analyses revealed that the carefully designed core–shell structures not only lead to a remarkable HAE effect, but also lower the oxidation energy barrier and enhance the selectivity of the direct pathway, resulting in the improved FAO catalytic activity.

## 2 Results and Discussion

**Figure 1:**
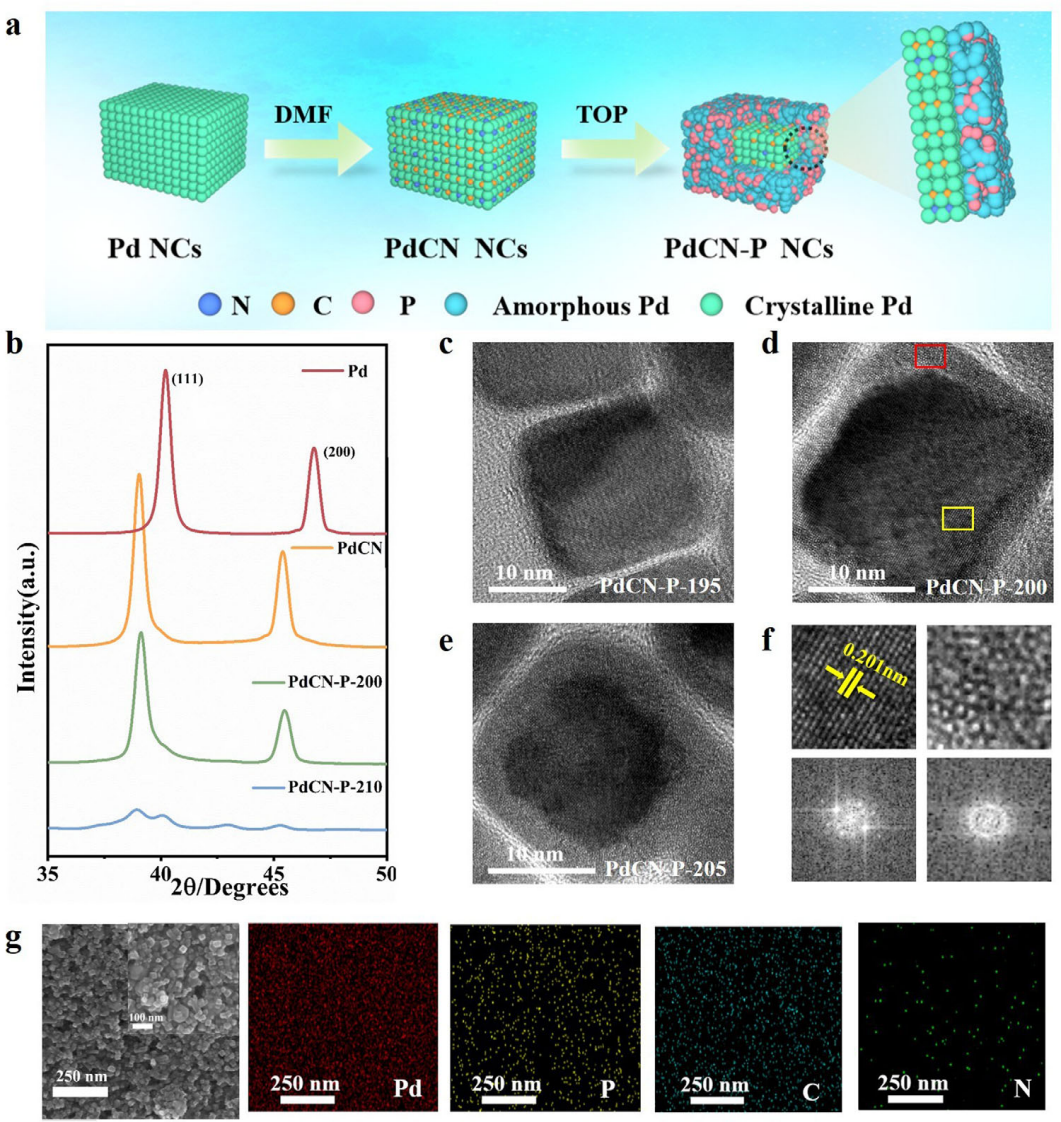
a) Schematic illustration of the synthesis process of PdCN‐P NCs, b) XRD spectra of Pd, PdCN, PdCN‐P‐200, and PdCN‐P‐210 NCs. HRTEM images of the c) PdCN‐P‐195 NCs, d) PdCN‐P‐200 NCs and e) PdCN‐P‐205 NCs. f) Magnified view and corresponding Fourier transform images of the crystalline and amorphous regions in (d), marked by yellow and red wireframes, respectively. g) SEM images and corresponding elemental mapping of PdCN‐P‐200 NCs, with the inset showing a magnified view.

In this work, we developed a three‐step method based on non‐metallic element doping to construct crystalline@amorphous core–shell Pd‐based NCs (**Figure** **1**a). First, pure Pd NCs were synthesized through modifying the method reported by Xia et al.^[^ ^11^ ^]^ The synthesized Pd NCs have a face‐centered cubic lattice with great monodispersity and crystallinity (Figure 1b; Figures S1 and S2a,c, Supporting Information). Then, the interstitial doping of C and N in Pd NCs (PdCN NCs) was achieved through a solvothermal method involving the use of N, N‐Dimethylformamide.^[^ ^12^ ^]^ X‐ray diffraction (XRD) pattern show that the characteristic diffraction peaks of the obtained PdCN NCs shifted to lower angles compared to those of Pd NCs, which confirms the successful incorporation of C and N (Figure 1b; Figure S1, Supporting Information). High resolution transmission electron microscope (HRTEM) images show that the PdCN NCs maintain excellent monodispersity and crystallinit (Figure S2b,d, Supporting Information). Finally, surface phosphating of the PdCN NCs was achieved through co‐heating with trioctylphosphine, resulting in well‐defined crystalline@amorphous core–shell PdCN‐P NCs.^[^ ^10^ ^]^ The ratio of P doping and the thickness of the PdP amorphous shell directly relies on the phosphating temperature (Figure 1c–e; Figure S3; Table S1, Supporting Information).Specifically, at phosphating temperatures of 195, 200, and 205, the amorphous shell thickness of PdCN‐P‐T NCs (T = 195, 200, 205) reached 1.5, 2.8, and 3.9 nm, respectively (Figure S4, Supporting Information). HRTEM images show that the core region of PdCN‐P‐200 sample has a clear 0.201 nm lattice streaks, which can be considered as the (200) crystal plane of PdCN crystals. Meanwhile the Fast Fourier transform patterns of the core and shell regions reveal an ordered crystalline structure and a disordered amorphous structure, respectively (Figure 1f; Figure S5, Supporting Information). Scanning electron microscopy (SEM) and the corresponding elemental distribution maps further confirmed the successfully doping of phosphorus in PdCN‐P samples (Figure 1g). As phosphating temperature reached 210 °C, the KirKendall effect becomes appreciable, resulting in a hollow structure with only a 6.1 nm amorphous shell of PdCN‐P‐210 NCs (Figures S4 and S6, Supporting Information). In contrast, attempts to directly construct crystalline@amorphous core–shell structures from Pd NCs resulted in a mixture of amorphous (PdP) and crystalline (Pd) particles at carefully controlled reaction temperatures (Figure S7, Supporting Information). Obviously, C and N doping can enhance the structure stability of Pd NCs and slow their amorphization kinetics, facilitating the formation of crystalline@amorphous core–shell structures.

**Figure 2:**
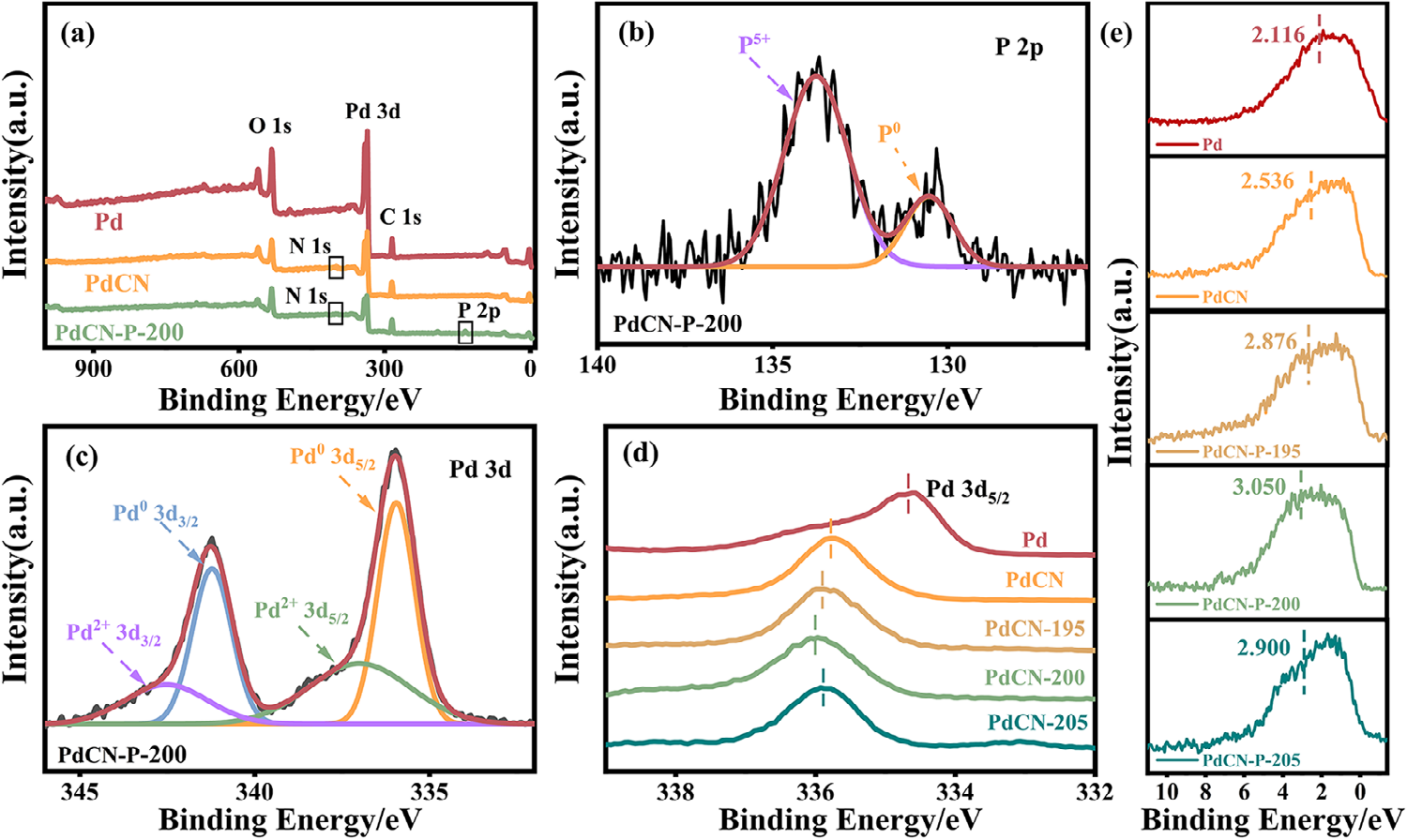
a) Survey XPS spectra of the PdCN‐P‐200 NCs. High‐resolution XPS spectra of b) P 2p and c) Pd 3d in PdCN‐P‐200 NCs. d) The Pd 3d high‐resolution XPS spectra and e) the XPS valence band spectra for the Pd, PdCN, PdCN‐P‐195, PdCN‐P‐200, and PdCN‐P‐205 NCs.

X‐ray Photoelectron Spectroscopy (XPS) was conducted to investigate the surface electronic structure information of PdCN‐P NCs. The XPS survey spectra confirm the presence of Pd, N, and P in PdCN‐P NCs (**Figure** **2**a). High‐resolution XPS P 2p spectra of PdCN‐P NCs show the typical binding peaks of P^5+^ and P^0^ at about 133.8 and 130.6 eV, respectively (Figure 2b; Figure S8, Supporting Information). The binding energy of P^0^ shifts positively compared to the standard value, indicating the reduction of electron density surrounding P atoms in the amorphous shell.^[^ ^13^ ^]^ The high‐resolution XPS Pd 3d signal of PdCN‐P NCs exhibits the Pd^0^ and Pd^2+^ species at ≈ 335.9/341.3 eV and 337.1/342.6 eV, respectively (Figure 2c; Figure S9, Supporting Information). Compared to the initial PdCN NCs, the binding energy of Pd^0^ in PdCN‐P‐T (T = 195, 200, 205) exhibits a positive shift, which reveals the reduction of electron density surrounding the surface Pd atoms (Figure 2d). Obviously, the reduction in electron density around Pd and P cannot be ascribed to electron transfer between these two elements, but rather to charge transfer from the amorphous shell to the crystalline core.^[^ ^14^ ^]^ The theoretical calculation results (Figure S10, Supporting Information) and the higher work function (Figure S11, Supporting Information) of the PdCN‐P‐200 compare to PdCN and PdP further confirm our conclusion.The decrease in surface charge density can lower the energy barrier for oxidation reactions, which accelerates the oxidation process of formic acid. As for the PdCN‐P‐210 NCs with hollow structure, the binding energy of Pd^0^ shows a negative shift relative to that of PdCN‐P‐200, which is attributed to the disappearance of the crystalline core (Figure S12, Supporting Information). Additionally, the formation of crystalline@amorphous core–shell structures also leads to the downward shifts of the d‐band center for PdCN‐P‐T (T = 195, 200, 205) (Figure 2e; Figure S13, Supporting Information). According to previous research, the regulation of the d‐band center can alter the adsorption energies of reactants and intermediate products, thereby influencing the FAO reaction pathway.^[^ ^15^ ^]^

**Figure 3:**
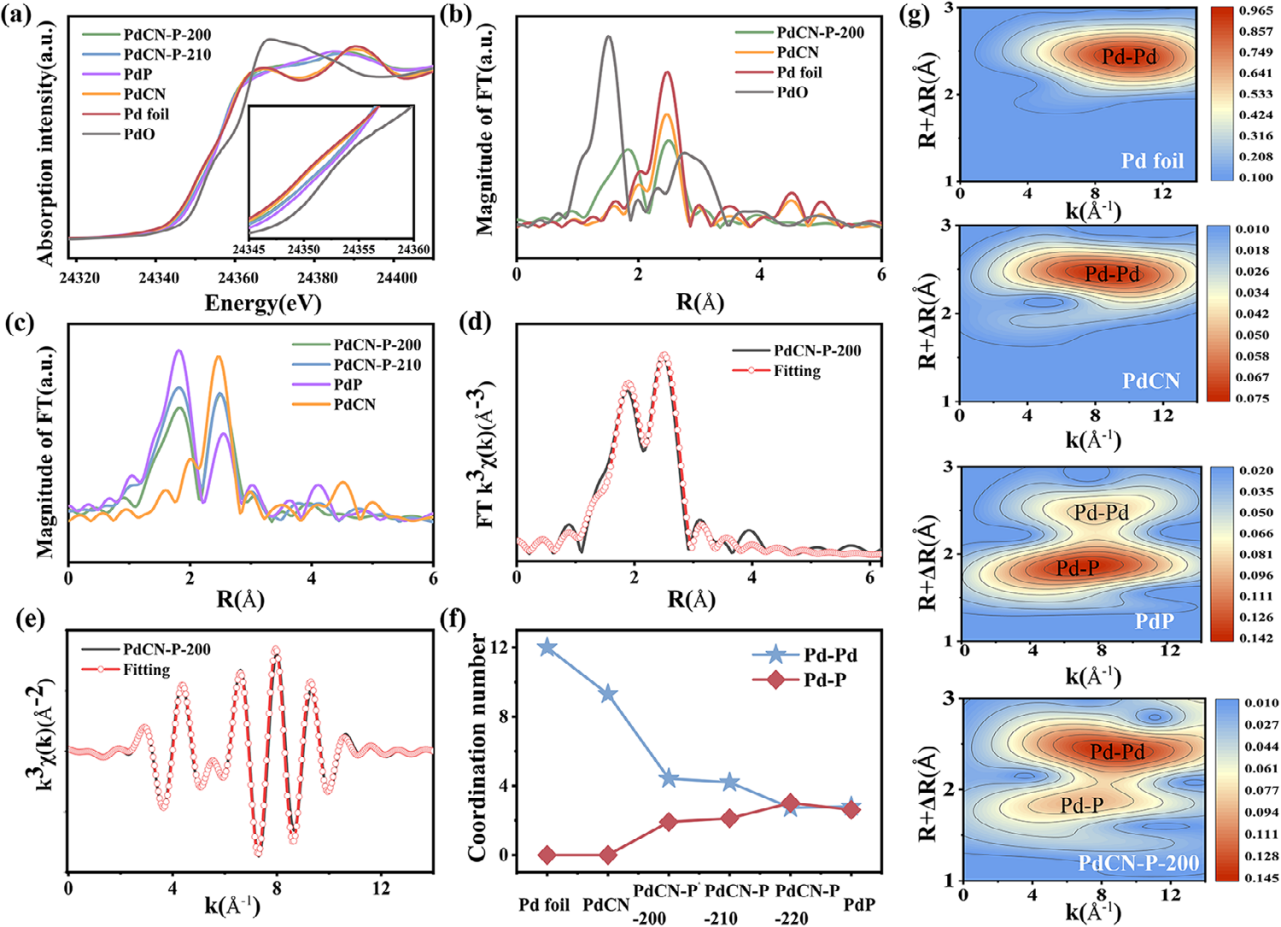
a) The Pd K‐edge XANES spectra of the PdCN‐P‐200 NCs, PdCN‐P‐210 NCs, PdP NCs, PdCN NCs, PdO, and Pd foil. b) The Pd K‐edge Fourier transform EXAFS spectra of the PdCN‐P‐200 NCs, PdCN NCs, PdO, and Pd foil. c) The Pd K‐edge Fourier transform EXAFS spectra of the PdCN‐P‐200 NCs, PdCN‐P‐210 NCs, PdP NCs, and PdCN NCs. The EXAFS fitting at (d) R space and (e) K space spectra of the PdCN‐P‐200 NCs. f) The coordination number of Pd─Pd bonds and Pd─P bonds in all samples extracted from EXAFS fitting. g) Pd K‐edge wavelet transform contour plots of the Pd foil, PdCN NCs, PdP NCs, and PdCN‐P‐200 NCs.

X‐ray absorption spectroscopy (XAS) was further used to analyze the coordination structures of Pd and P species in the obtained samples. The X‐ray absorption nearedge structure (XANES) spectra of Pd in PdCN NCs show similar features to Pd foil, implying that the Pd in PdCN NCs is mainly in the metallic state (**Figure** **3**a). The absorption edge of PdCN‐P‐T (T = 200 and 210) is located between Pd foil and PdO, which indicates an increased oxidation state of Pd. These observations are consistent with the XPS analysis described above. Figure 3b presents the Fourier‐transformed extended X‐ray absorption fine structure (EXAFS) spectra of PdCN, the peak observed at ≈2.5 Å corresponds to the Pd–Pd coordination. Corresponding Pd–Pd peak is significantly weakened in PdCN‐P‐200 sample, which reflects the lower coordination number. Moreover, the characteristic peak Pd–P coordination (at 1.8 Å) appears in PdCN‐P‐200 sample. The decrease in the Pd–Pd coordination number, along with the appearance of Pd–P coordination, further confirms the formation of an amorphous shell layer on the surface of the PdCN‐P‐200 sample. Moreover, as for PdCN‐P‐210 and ‐220, the characteristic peaks of Pd‐P become stronger than that of PdCN‐P‐200 sample due to the further phosphating (Figure 3c).

Quantitative EXAFS fitting was involved to get more insight on the local chemical coordination environment around the Pd atom in the samples (Figure 3d,e; Figures S14–S17, Supporting Information). The coordination numbers of Pd‐Pd bonds for PdCN, PdCN‐P‐200, and PdCN‐P‐210 are 9.31, 4.42, and 4.20, respectively (Figure 3f; Table S2, Supporting Information). The decrease in Pd–Pd coordination number reveals an increase in the PdP amorphous region on the surface. Notably, PdCN‐P‐200 (crystalline@amorphous core–shell structures), PdCN‐P‐210 (hollow structure), and PdCN‐P‐220 (hollow structure) exhibit Pd‐P coordination numbers of 1.91, 2.12, and 3.01, respectively. The rapid increase in the Pd‐P coordination number following the disappearance of crystal core further supports the notion that interstitial doping of C and N slows the amorphization kinetics of Pd‐based NCs. Wavelet transform contour plots are shown in Figure 3g and Figures S15 and S16 (Supporting Information), as for the PdCN NCs sample, only one intensity maximum was observed at ≈8 Å^−1^ corresponding to Pd–Pd. The rest of the samples show another intensity maximum at ≈6.5 Å^−1^, which corresponds to Pd‐P.

Overall, through phosphorization of PdCN nanocrystals, we have synthesized PdCN‐P NCs with crystalline@amorphous core–shell structures. TEM and XAS analyses indicate that interstitial doping of C and N effectively slows down the amorphization kinetics of Pd‐based NCs, allowing us for precise control over the thickness of amorphous shell for PdCN‐P NCs. In addition, the PdCN‐P NCs exhibit reduced surface electron density and a lower d‐band center, which can lower the oxidation barrier and optimize the reaction pathway, thereby facilitating FAO.

**Figure 4:**
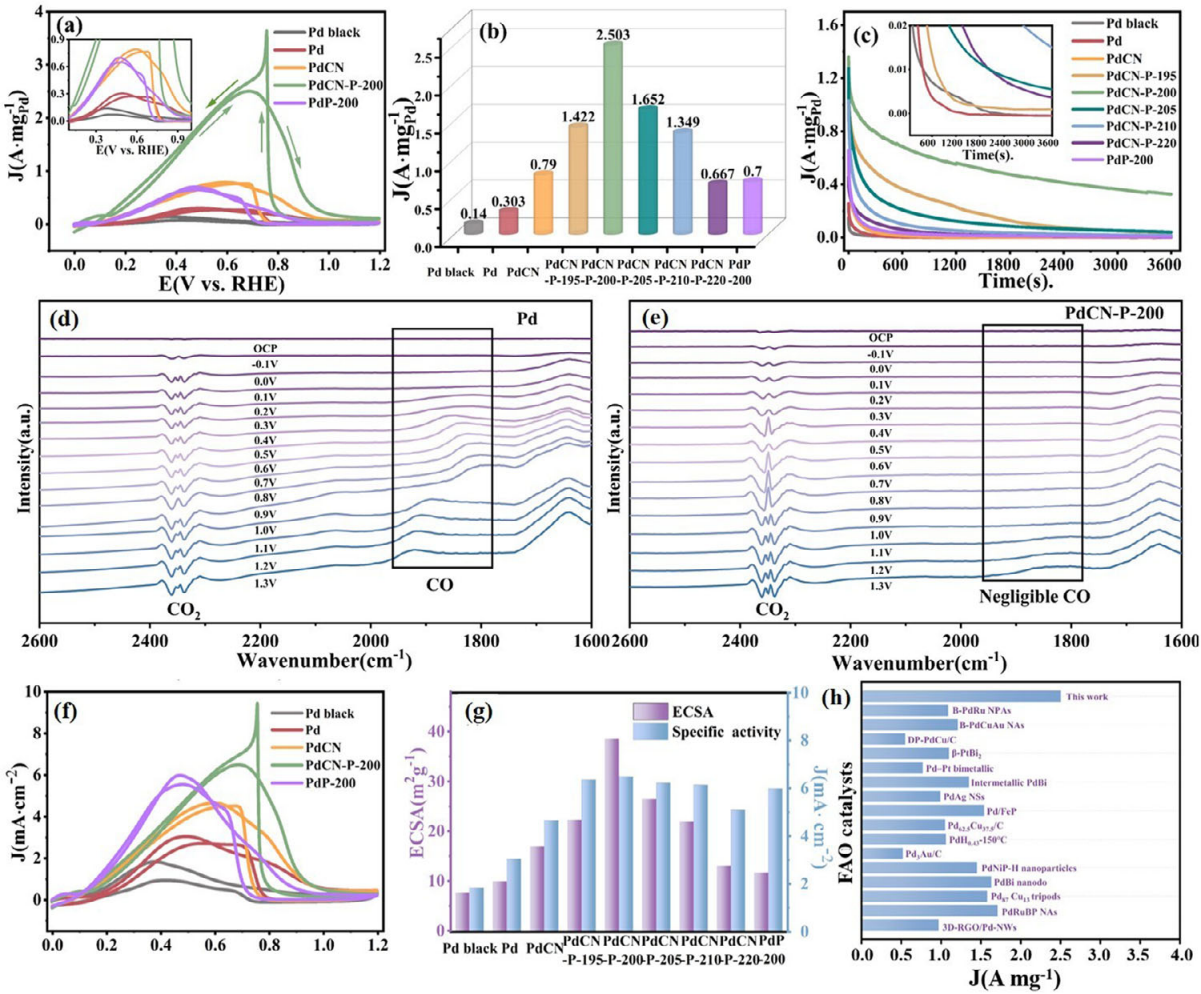
a)The mass‐normalized CV curves of the Pd black, Pd, PdCN, PdCN‐P‐200, and PdP catalysts in a 0.5 m H_2_SO_4_ solution containing 0.5 m HCOOH. b) The mass activity values and c) chronoamperometric curves for 3600 s of all catalysts. The in situ ATR‐FTIR spectra of d)Pd and e)PdCN‐P‐200 at different potentials for FAO. f) The ECSA‐normalized CV curves of the Pd black, Pd, PdCN, PdCN‐P‐200, and PdP catalysts in the 0.5 m H_2_SO_4_ solution containing 0.5 m HCOOH. g) The ECSA and specific activity values of all catalysts. g)Comparison of PdCN‐P‐200 and some reported palladium‐based catalysts in terms of mass activity for formic acid oxidation. The inset in (a) and (c) show the local magnification of corresponding curve.

To evaluate the FAO electrocatalytic performance of PdCN‐P‐T (T = 195, 200, 205, 210, 220), we performed electrochemical measurements using a standard three‐electrode setup in a solution containing 0.5 m H₂SO₄ and 0.5 m HCOOH at room temperature. For comparison, several samples (including Pd black, Pd, PdCN, and PdP NCs) were also measured under the same test conditions. The FAO mass activities of all catalysts were obtained by normalizing the peak current of the cyclic voltammetry (CV) results by the total amount of Pd loaded on the working electrode (**Figure** **4**a,b; Figure S18a, Supporting Information). The Pd contents in the catalysts were determined by ICP‐MS (Table S3, Supporting Information). The PdCN‐P‐200 sample exhibits the highest FAO activity with the mass activity of 2.503 A mg⁻¹_Pd_, which value is 17.88, 8.26, 3.19, and 3.58 times higher than that of Pd black, Pd NCs, PdCN NCs, and PdP NCs, respectively. To confirm the reliability of the catalytic activity data, we repeated the experiment 10 times. The results in Figure S19 (Supporting Information) shows consistent catalytic activity across all trials. Meanwhile, PdCN‐P‐200 also exhibit the best catalytic stability among all samples during chronoamperometric (CA) tests (Figure 4c). After 3600s of CA testing, PdCN‐P‐200 retained the mass activity of 0.326 A mg⁻¹_Pd_. After ≈4 h, commercial Pd black was nearly completely deactivated, while PdCN‐P‐200 was still not completely deactivated at 15 h. These results highlight the exceptional stability of PdCN‐P‐200 (Figure S20, Supporting Information). This excellent catalytic performance of FAO can be partially attributed to the selectivity of PdCN‐P‐200 for FAO pathway. According to the CV measurements in a solution containing 0.5 m H₂SO₄ and 0.5 m HCOOH, the first peak during the forward scan corresponds to the direct oxidation of HCOOH to CO_2_, while the second peak is associated with the indirect FAO pathway to form CO.^[^ ^16^ ^]^ The second peak for PdCN‐P‐200 sample is quite weak, which indicates its high preference for the direct pathway (Figure 4a). The high preference for direct pathway can mitigate CO poisoning of PdCN‐P‐200, thereby enhancing its catalytic stability.

In situ attenuated total reflectance Fourier transform infrared spectroscopy (ATR‐FTIR) was employed to investigate the pathway selectivity of PdCN‐P‐200 and Pd when catalyzing FAO, providing insights into the reaction pathway at the molecular level. As show in Figure 4d,e, the in situ ATR‐FTIR results reveal that both CO₂ and CO vibrational peaks are present on the Pd surface during FAO, indicating that the reaction primarily proceeds via a dual‐pathway mechanism. As for PdCN‐P‐200, a distinct CO₂ vibrational peak is observed, while only a negligible CO peak appears. This suggests that PdCN‐P‐200 exhibits a strong preference for the direct FAO pathway, consistent with the electrochemical testing results.

Additionally, we assessed the ECSA of the sample as an indicator of the density of active sites on the catalyst surface. CV measurements were carried out in 0.5 m H_2_SO_4_ with N_2_ saturation at a scan rate of 50 mV s^−1^ to determine the ECSA of the catalysts in electrochemical environments. Details of the calculation method are provided in the supporting information. The results indicate that the ECSA of PdCN‐P‐200 is of up to 39.96 m^2^ g^−1^_Pd_, which is 5.05, 3.88, 2.27and 3.29 times greater than that value of Pd black (7.91 m^2^ g^−1^_Pd_), Pd NCs (10.30 m^2^ g^−1^_Pd_), PdCN (17.60 m^2^ g^−1^_Pd_) and PdP (12.13 m^2^ g^−1^_Pd_) (Figure 4g; Figure S21, Supporting Information).In the previous discussion, we highlighted that the phosphating temperature can effectively regulate the amorphous shell thickness of PdCN‐P‐T samples. These adjustable amorphous shells can influence the geometry and electronic structure of the catalytic sites, thereby altering HAE in PdCN‐P‐T samples.^[^ ^9^, ^17^ ^]^ To investigate this impact, we further measured the ECSA of PdCN‐P‐T (T = 195, 205, 210, 220). The results show that the ECSA of the PdCN‐P‐T samples demonstrates a distinct “volcano” trend with increasing phosphating temperature and PdCN‐P‐200 shows the highest ECSA (Figure 4g; Figure S21, Supporting Information). Obviously, at a phosphating temperature of 200 °C, the PdCN‐P system with a 2.8 nm amorphous shell achieves the optimal HAE required for FAO.

Finally, the specific activity, which is a good indicator for the intrinsic FAO activity, are obtained by normalizing the peak current of the CV test by ECSA (Figure 4f,g; Figure S18b, Supporting Information).^[^ ^18^ ^]^ PdCN‐P‐200 exhibits the highest specific activity of 6.265 mA cm^−2^, which value is 3.54, 2.13, and 1.40 times higher than that of Pd black (1.771 mA cm^−2^), Pd NCs (2.941 mA cm^−2^) and PdCN NCs (4.491 mA cm^−2^), respectively. It is suggested that P doping can enhance the adsorption strength of HCOOH on the surface of Pd‐based materials, thereby improving the catalytic site activity.^[^ ^19^ ^]^ Given the similar specific activities of PdP and PdCN‐P‐T (195, 200, 205, 210), we attribute the enhanced specific activity of PdCN‐P‐T(195, 200, 205, 210) predominantly to the P doping. On the other hand, the charge transfer between the core–shell heterojunction reduces the surface charge density of PdCN‐P‐200 NCs, thereby lowering the oxidation barrier. As a result, the specific activity of PdCN‐P‐200 is slightly higher than that of PdCN‐P‐195, ‐205, and ‐210 NCs.

In summary, PdCN‐P‐200 exhibits the best catalytic performance for FAO, with a mass activity of up to 2.503 A mg⁻¹_Pd_. The exceptional catalytic activity and stability of PdCN‐P‐200 position it as one of the most remarkable FAO catalysts reported to date (Figure 4h; Table S4, Supporting Information).^[^ ^20^ ^]^ This excellent catalytic performance is primarily attributed to three factors: First, the doping of non‐metallic elements optimized the adsorption of FAO intermediates on the catalyst surface, enhanced the direct oxidation pathway preference of PdCN‐P‐200 for FAO process, mitigated CO poisoning, and improved the catalytic stability. Second, the carefully optimized amorphous shell thickness in PdCN‐P‐200 NCs lead to a remarkable HAE effect, which significantly increases its ECSA. Third, P doping together with charge transfer between the core–shell heterojunction both strengthen the adsorption of HCOOH and reduce the oxidation barrier of FAO, thereby improving the specific activity of the catalytic sites.

**Figure 5:**
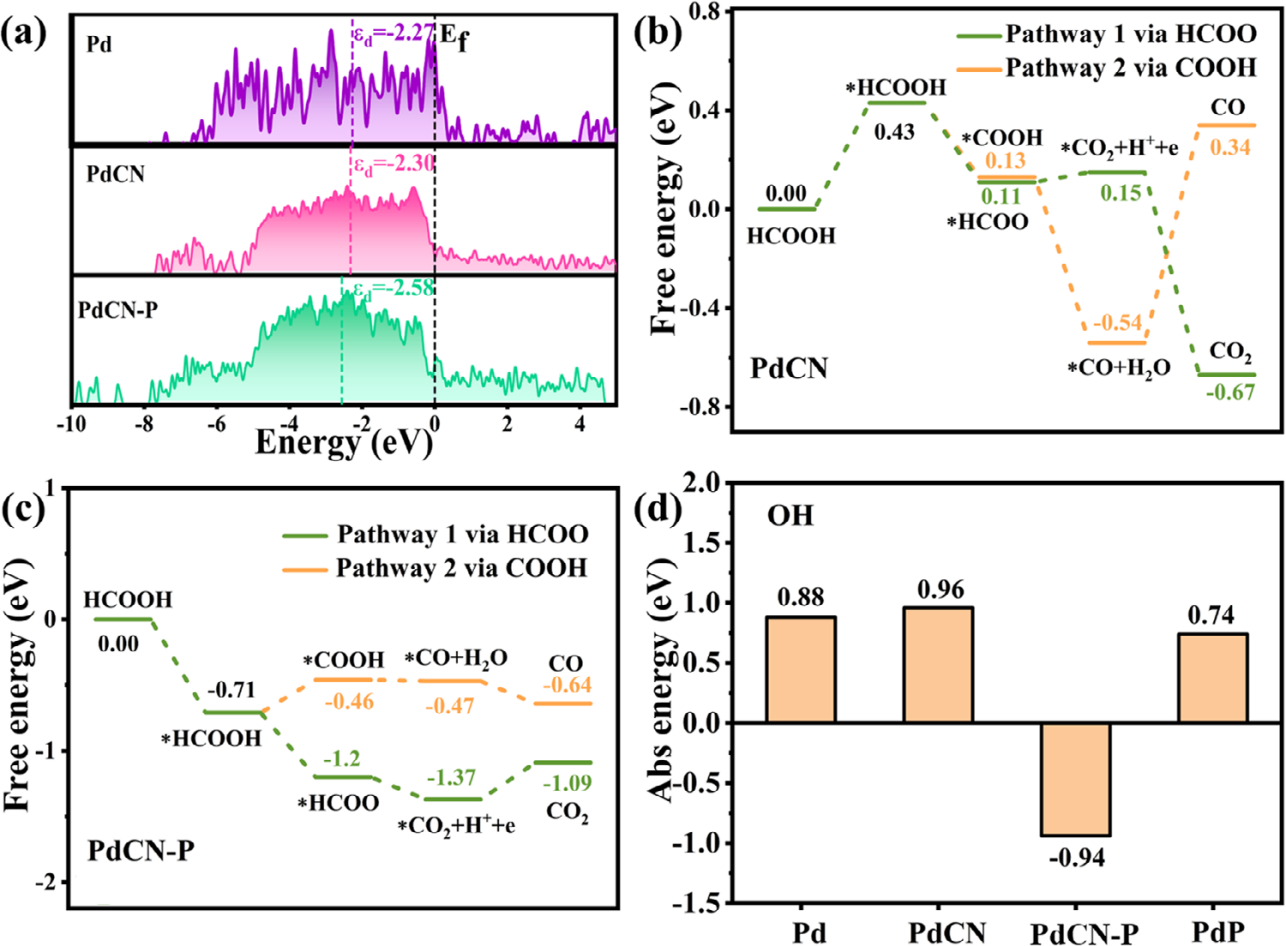
a) Calculated pDOS of Pd, PdCN, and PdCN‐P catalysts. The black dashed line denotes the position of the Fermi level. The reaction pathways of b) PdCN and c) PdCN‐P catalysts for FAO. d) Adsorption energy of ^*^OH on Pd, PdCN, PdCN‐P, and PdP catalysts.

To gain deeper insight in the mechanism of FAO on Pd‐based catalysts, DFT calculations were performed using multilayer models for Pd (111), PdCN, PdCN‐P and PdP (Figuers S22–25). First, we calculated the projected densities of states (pDOS) for the d orbits of Pd (111), PdCN, and PdCN‐P models (**Figure** **5**a). The results revealed that the d‐band centers of Pd (111), PdCN, and PdCN‐P were found to be −2.27, −2.30, and −2.58 eV, respectively. These findings suggest that doping with carbon (C), nitrogen (N), and phosphorus (P) leads to a decrease in the d‐band center of the catalyst, which aligns with the XPS spectra we obtained in experiments.

Further, the free energy changes (ΔG) of all FAO elementary steps were calculated to investigate the catalytic performance of these Pd‐based catalysts (Figure 5b,c; Figures S26 and S27, Supporting Information). In general, depending on the presence of CO^*^ intermediate, FAO involves two reaction mechanisms—direct and indirect. Both mechanisms were considered in this study. The results show that the adsorption of HCOOH on Pd and PdCN to form the *HCOOH intermediate requires energy inputs of 0.45 and 0.43 eV, respectively (Figure 5b; Figure S26, Supporting Information). In contrast, the initial adsorption of HCOOH on PdCN‐P and PdP catalysts is exothermic, indicating that the entire catalytic reaction process is more readily activated on PdCN‐P and PdP. Additionally, to complete the entire direct oxidation process, the PdCN‐P catalyst requires a minimum input energy of 0.28 eV, which values are 0.45, 0.43, and 0.43 eV for Pd, PdCN, and PdP, respectively. Faster activation kinetics and lower input energy can effectively enhance the activity of catalytic site. Therefore, in electrochemical testing, the PdCN‐P‐200 sample exhibits the highest specific activity.

Finally, CO poisoning needs to be avoided during the FAO process, as CO can occupy the catalyst sites and hinder further oxidation of formic acid. This requires the ability to oxidize surface CO while improving the catalyst's preference for direct pathways. The selectivity of FAO catalysts for direct or indirect pathways is influenced by the difference in ΔG between steps *COOH and *HCOO. The exceedingly small ΔG difference between *COOH and *HCOO for both Pd (0.01 eV) and PdCN (0.02 eV) allows the indirect oxidation pathway of HCOOH, leading to CO generation which reduce the stability of the catalyst. Conversely, for PdCN‐P, a ΔG difference of ≈0.74 eV between *COOH and *HCOO inhibits the indirect oxidation pathway. This result is consistent with the in situ ATR‐FTIR characterization results, which shows that PdCN‐P‐200 generates a prominent CO_2_ signal and a negligible CO signal during the FAO process. Further, considering that the surface‐adsorbed *OH can efficiently oxidize CO to CO_2_, thereby mitigating CO poisoning and enhancing catalyst stability.^[^ ^18a^ ^]^ We calculated the adsorption energy of *OH on the surface of all models (Figure 5d; Figure S28, Supporting Information). The calculated *OH adsorption energies (E_ads_) values for Pd (111), PdCN, PdCN‐P, and PdP catalysts are 0.88, 0.96, −0.94, and 0.74 eV, respectively. These results indicate that the absorbed *CO on PdCN‐P catalysts can be easily oxidized to CO_2_. Combined with its preference for the direct oxidation pathway, this suggests that PdCN‐P exhibits the highest CO resistance among the calculated models, consistent with the results obtained from the CO‐stripping experiments and CA testing (Figures S20 and S29, Supporting Information).

## 3 Conclusion

In summary, we have demonstrated non‐metallic element doping as an effective approach for the synthesis and precise control of Pd‐based crystalline@amorphous core–shell structures. The obtained PdCN‐P samples exhibit the shell‐thickness‐dependent catalytic performance, and the PdCN‐P‐200 sample with an amorphous shell thickness of 2.8 nm possesses the highest mass activity (2.503 A mg^−1^_Pd_) for FAO. Detailed experimental and theoretical analyses reveal that the carefully optimized thickness of the amorphous shell in PdCN‐P NCs can lead to a remarkable HAE effect, which significantly increases its ECSA. Moreover, P doping together with the charge transfer at core–shell heterojunction can lower the FAO reaction barrier and enhance the adsorption strength of formic acid, thus enhancing the specific activity of crystalline@amorphous core–shell PdCN‐P NCs. Lastly, the formation of crystalline‐amorphous heterojunctions can optimize the adsorption strength of FAO intermediates and enhances the selectivity for the direct oxidation pathway of FAO. These findings highlight the remarkable potential of crystalline@amorphous core–shell structures in electrocatalysis and represent a significant step toward the commercialization of DFAFCs.

## Conflict of Interest

The authors declare no conflict of interest.

## Supporting information



Supporting Information

## Data Availability

The data that support the findings of this study are available from the corresponding author upon reasonable request.
