# Supplementary material for: Constructing Palladium‐Based Crystalline@Amorphous Core–Shell Heterojunctions for Efficient Formic Acid Oxidation
Source: Adv Sci (Weinh). 2025 Apr 30;12(27):2504469. doi: 10.1002/advs.202504469 (PMC12279218; doi:10.1002/advs.202504469)
Supplement: Supplementary file 1 — Supporting Information [file ADVS-12-2504469-s001.docx]

**Supporting Information**

**Constructing Palladium-Based Crystalline@Amorphous Core-Shell Heterojunctions for Efficient Formic Acid Oxidation**

*Huiling Li,^[1]^ Jingkun Yu,^[2]^ Yongming Sui*^[1]^, Weibin Wang,^[1]^ Jiewen Liu,^[1]^ Libo Sheng,^[1]^ Ankang Chen,^[1]^ Siyu Lu*^[2]^ and Bo Zou*^[1]^*

Dedication: Huiling Li and Jingkun Yu contributed equally to this work.

H.Li, W. Wang, J. Liu, L. Sheng, A. Chen, Prof. Y. Sui, Prof. B Zou
State Key Laboratory of High Pressure and Superhard Materials

College of Physics

Jilin University
Changchun 130012, China
E-mail: suiym@jlu.edu.cn; zoubo@jlu.edu.cn.

J.Yu, Prof. S. Lu
College of Chemistry and Pingyuan Laboratory

Zhengzhou University

Zhengzhou 45000, China

E-mail: sylu2013@zzu.edu.cn

1. **Experimental**

**1.1 Chemicals and Materials.** Na_2_PdCl_4_, (99.99%), L-ascorbic acid (99.99%), Pd black (99.95%) Isopropyl Alcohol (99.9%), Oleylamine (OAm) (80-90%), Formic acid (88%) were purchased from aladdin. N, N-Dimethylformamide (DMF) (99.5%), Acetone (99.5%), PVP (Mw ≈ 55,000), KBr, Tri-n-octylphosphine (TOP) (90%), Nafion solution (5%) were purchased from Titan. All the chemicals were used as received without further purification. All aqueous solutions were prepared using Milli-Q water with a resistivity of 18.2 MΩ·cm at room temperature.

**1.2 Synthesis of Pd nanocubes.** Palladium nanocubes were synthesized by modifying the synthesis method reported by Xia,^[1]^ 1.05 g of polyvinyl pyrrolidone (PVP, MW ~55000), 0.6 g of L-ascorbic acid (AA) and 6 g of KBr were dissolved in 80 mL of deionized water in a 250 ml vial. The mixture was then heated in an oil bath under magnetic stirring at 80 °C for 10 minutes. Subsequently, the reaction solution was then maintained at 80 °C for 3 hours after 30 mL of deionized water solution containing 570 mg of Na_2_PdCl_4_ was injected. Finally, the Pd nanocubes were collected by centrifugation and washed with water, ethanol and acetone for several times.

**1.3 Synthesis of PdCN nanocubes.** PdCN nanocubes were synthesized by modifying the synthesis method reported by Fan,^[2]^ 15 mg prepared Pd nanocubes were dispersed in 30 mL DMF, poured into a Teflon-lined stainles steel autoclave. The sealed vessel was heated from room temperature to 180°C, maintained at this temperature for 2 h and then allowed to cool naturally to room temperature. The product was then centrifuged at 10000 rpm for 15 min, washed three times with ethanol and dried at room temperature.

**1.4 Synthesis of PdCN-P-T (T= 195, 200, 205, 210 and 220) and PdP.** The method of doping P is referred to the synthesis method reported by Jin,^[3]^ 21mg PdCN nanocubes were mixed with 77 mL of OAm, into which 7 mL of TOP was added. The mixture was then heated at 195, 200, 205, 210 and 220°C for 10 min to achieve PdCN-P-T (T= 195, 200, 205, 210 and 220). In addition, 21 mg of Pd nanocubes were mixed with 77 ml of OAm and then 7 ml of TOP was added. The mixture was then heated at 195°C for 10 minutes to obtain a mixture of amorphous (PdP) and crystalline (Pd) particles. After changing the temperature to 200°C, pure amorphous PdP nanoparticles were obtained.

**1.5 Characterization.** The phase structure of all samples was characterized by a Rigaku X-ray diffraction (XRD, R-AXIS-RAPIDII, Cu K α radiation, λ =1.5406 Å). The morphology, elemental and crystal characteristics of the samples were identified by transmission electron microscopy (TEM, JEM-2100F, JEOL) and scanning electron microscopy (SEM, Regulus 8100, Hitachi). The surface composition and metal values of samples were analyzed by X-ray photoelectron spectroscopy (XPS, ESCALAB-250Xi). The contents of Pd and P were determined by inductively coupled plasma mass spectrometry (ICP-MS, iCAP, RQ).

**1.6 Preparation of the working electrode.** First, 20 mg of Vulcan XC-72R carbon black were dispersed in 20 mL of ethanol and sonicated in an ice bath for 1 h to obtain a homogeneous solution. Subsequently, in a 10 mL glass vial, 4.05 mL of the carbon solution that had been prepared was mixed dropwise with the phosphated sample solution that contained 450 μg of Pd element, as measured by ICP-MS. This was followed by two hours of sonication in an ice bath. The obtained catalyst on carbon (catalyst/C, 10 wt%) was collected by centrifugation at 13000 rpm for 10 min, followed by washing five times with a mixed solvent of chloroform and ethanol (v:v = 1:1).^[4]^ Then, the obtained catalyst/C was finally dispersed in 3 mL isopropanol and the concentration of Pd in catalyst/C was further determined by ICP. Finally, 30 μL of Nafion alcohol solution (5 wt%) were added in the catalyst/C solution, followed by sonication for another 30 min to obtain catalyst ink. To guarantee a clean surface before use, glassy carbon electrode (GCE, 5mm) was polished by Al_2_O_3_ slurry with particle sizes of 300 and 50 nm, respectively, followed by washing with Milli-Q water and ethanol then the catalyst ink containing 3ug Pd element was dropped on its surface and dried at room temperature. For consistency, the prepared Pd black, Pd, PdCN and PdP working electrodes also contained 3ug of Pd element.

**1.7 Electrochemical measurements.** The electrochemical measurements in this work were carried out by a three-electrode cell using a CHI 660E electrochemical work station (Chenhua, China) at room temperature. A glassy carbon electrode (GCE, 5mm) with a catalyst coating serves as the working electrode. The Ag/AgCl electrode served as the reference electrode and the Pt plate as the counter electrode. All potentials were converted into the values in reference to reversible hydrogen electrode (RHE) according to E_(RHE)_ = E_(Ag/AgCl)_ + 0.197 + 0.059 × pH. The Cyclic voltammogram (CV) were measured in N_2_-saturated 0.5M H_2_SO_4_ solutions and N_2_-saturated solutions containing 0.5M H_2_SO_4_ and 0.5M HCOOH, respectively, with a potential range from -0.2 V to1 V (Vs. Ag/AgCl) at a scan rate of 50 mV∙ s^-1^. The chronoamperometric (CA) curves of various catalysts were measured in 0.5 M H_2_SO_4_ + 0.5 M HCOOH electrolyte at a fixed potential of 0.4 V versus RHE. The electrochemically active surface area (ECSA) was calculated based on the area of the reduction peak of PdO in the as-obtained CV curves. According to the equation:^[5]^

ECSA = Q/ (0.405 × m_Pd_)

estimate the ECSA values. Where m denotes the Pd mass on the working electrode surfaces. Q value represents the reduction charge of PdO. 0.405 mC cm^−2^ is a constant assuming that a monolayer of PdO is reduced on Pd surface.

**1.8 Statistical Analysis.** In our work, all of the thickness of amorphous shell layer were measured using the following method. Firstly, TEM images of all samples were obtained with a JEM-2200FS with an emission gun operating at 200 kV. Secondly, the TEM images were introduced into the Nano Measurer 1.2 software and the thickness of amorphous shell layer of the samples were calibrated. Thirdly, the calibrated the thickness of amorphous shell layer of the sample data were introduced into the Origin 2018 software and fitted with Gaussian Function to obtain the average thickness of amorphous shell layer of the sample. The measurement data were finally represented as the mean ± standard error (SE).

**1.9 Computational models and methods.** All density functional theory (DFT) computations were carried out by using the Vienna *Ab* *Initio* Simulation Package (VASP) with the projector-augmented wave (PAW) method and a cutoff energy of 450 eV was adopted.^[6]^ The exchange-correlation interactions were described by the Perdew–Burke–Ernzerhof (PBE) functional within the generalized gradient approximation (GGA).^[7]^ The convergence thresholds for energy and force were set to 10^−5^ eV and 0.03 eV Å^−1^, respectively. The Pd (111) surface was simulated with a 3×3 five-layer slab with the top three layers relaxed and the bottom two layers fixed in all calculations, in which a vacuum space of 15 Å in the z-direction was adopted to avoid the interactions between periodic images. The Brillouin zone was sampled with a Monkhorst–Pack grid of 3 × 3 × 1 for geometry optimization.^[8]^

According to our computations, H atoms tend to insert into the lattice gaps of Pd, while P atoms replace some of the Pd sites, which were thus chosen as the catalysts for formic acid oxidation. The adsorption energy (*E*_ads_) of OH on Pd-based catalysts was defined as *E*_ads_ = *E_OH_*_*_ − *E*_Slab_ − E*_H₂O_* + ^1^/_2_E*_H₂_*, where *E_OH_*_*_, *E*_Slab_, E*_H₂O_* and E*_H₂_* represent the total electronic energies of the adsorbed OH species, the pristine Pd-based catalysts, and the isolated H_2_O、H_2_ molecule, respectively. The Gibbs energy (G) of the intermediates was calculated by using the computational hydrogen electrode (CHE) model proposed by Nørskov et al. ^[9]^ The chemical potential of the H^+^/e^-^ pair in aqueous solution is related to that of half of the H_2_ gas molecule at standard hydrogen electrode (SHE) conditions. According to this method, the Gibbs energy change (ΔG) can be determined as follows: $\Delta G = \Delta E + \Delta ZPE - T\Delta S + {\Delta G}_{pH} + eU$, where $\Delta E$ is the reaction energy of the reactants and products adsorbed on the catalyst, calculated directly by DFT; $\Delta ZPE$ and $\Delta S$ are the changes in zero-point energy and entropy at 298.15 K, which can be calculated by the vibration frequency. ${\Delta G}_{pH}$ is the free energy correction of pH, which can be calculated by: ${\Delta G}_{pH}=k_{B}T \times pH \times ln10$.

The d-band center (ε_d_) calculated from the first moment as given by:

$$ℇ_{d}={\int_{-\infty}^{+\infty} \rho\left( E \right)\left( E-E_{F} \right)dE}/{\int_{-\infty}^{+\infty} \left( E-E_{F} \right)dE}$$

where ρ(E) is the DOS projected on the d-states of the metal atoms and E_F_ is the Fermi level of the system.^[10]^

1. **Supplementary figures and tables**


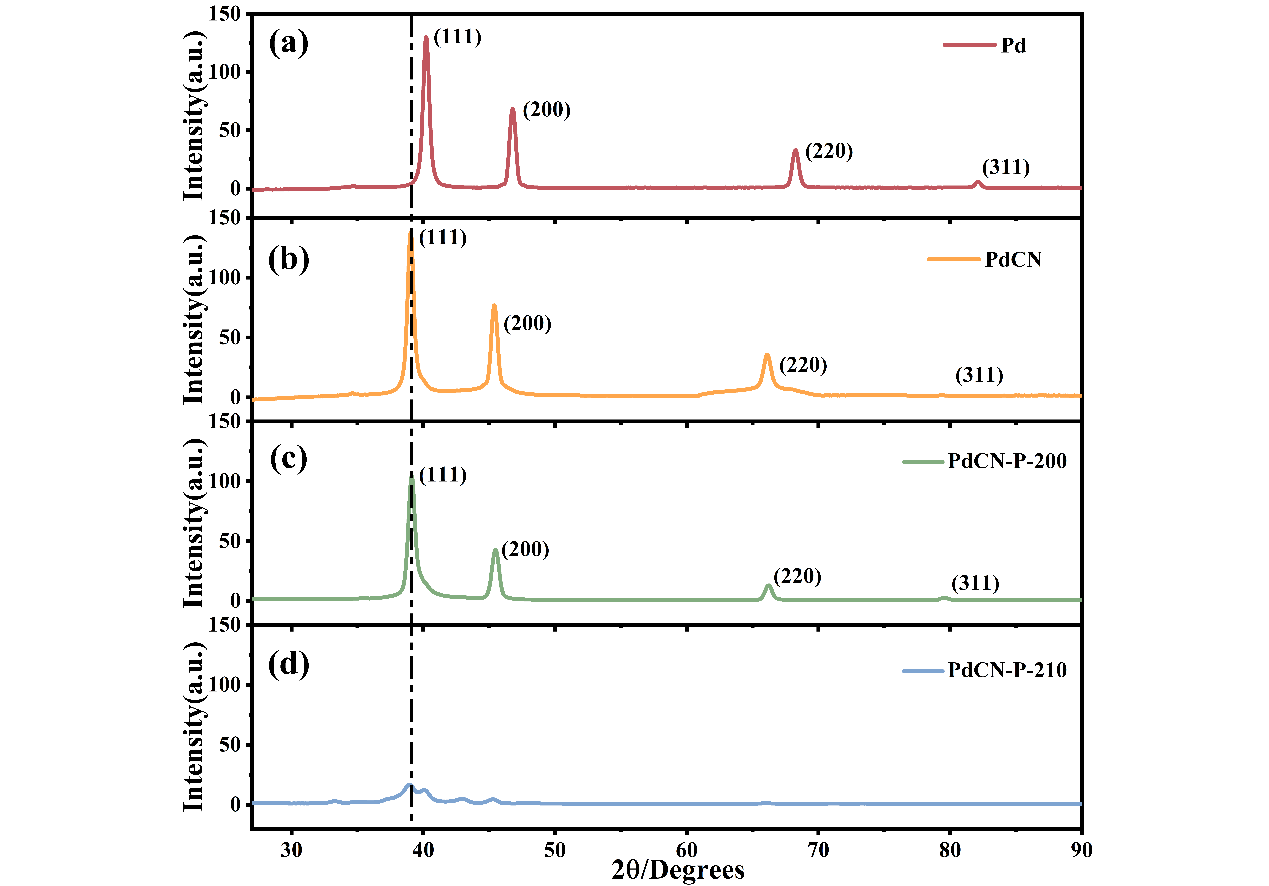


**Figure S1.** XRD spectra of Pd, PdCN, PdCN-P-200 and PdCN-P-210 NCs.


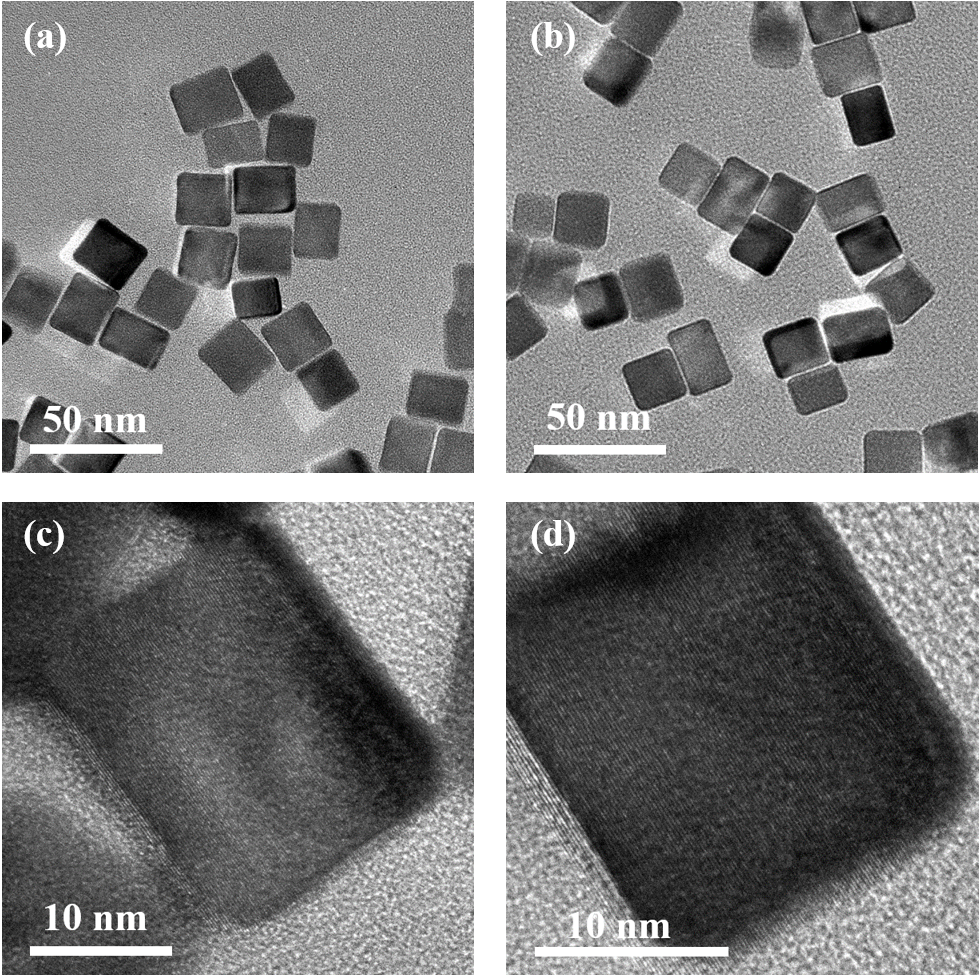


**Figure S2.** TEM and HRTEM images of (a, c) the Pd nanocubes and (b, d) the PdCN nanocubes.


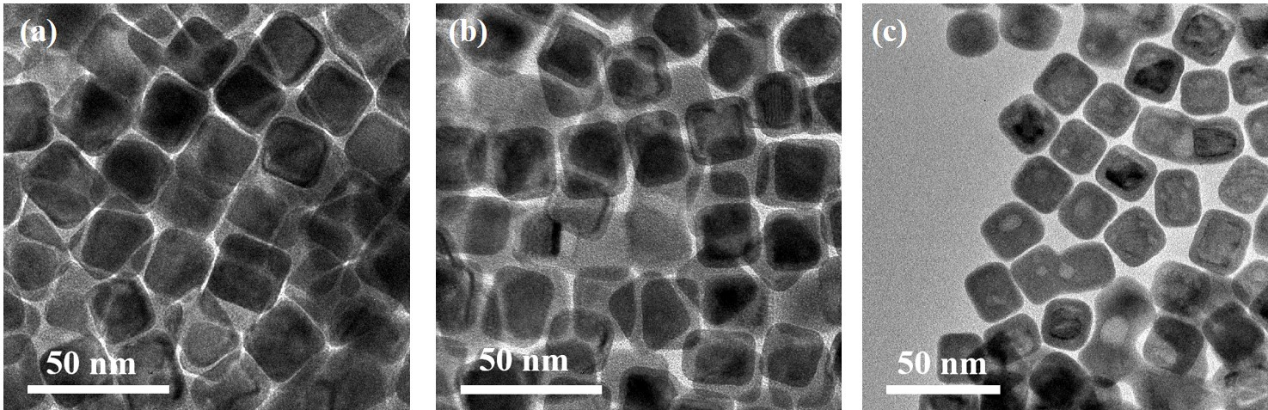


**Figure S3.**TEM images of the (a) PdCN-P-195 NCs, (b) PdCN-P-200 NCs and (c) PdCN-P-205 NCs.


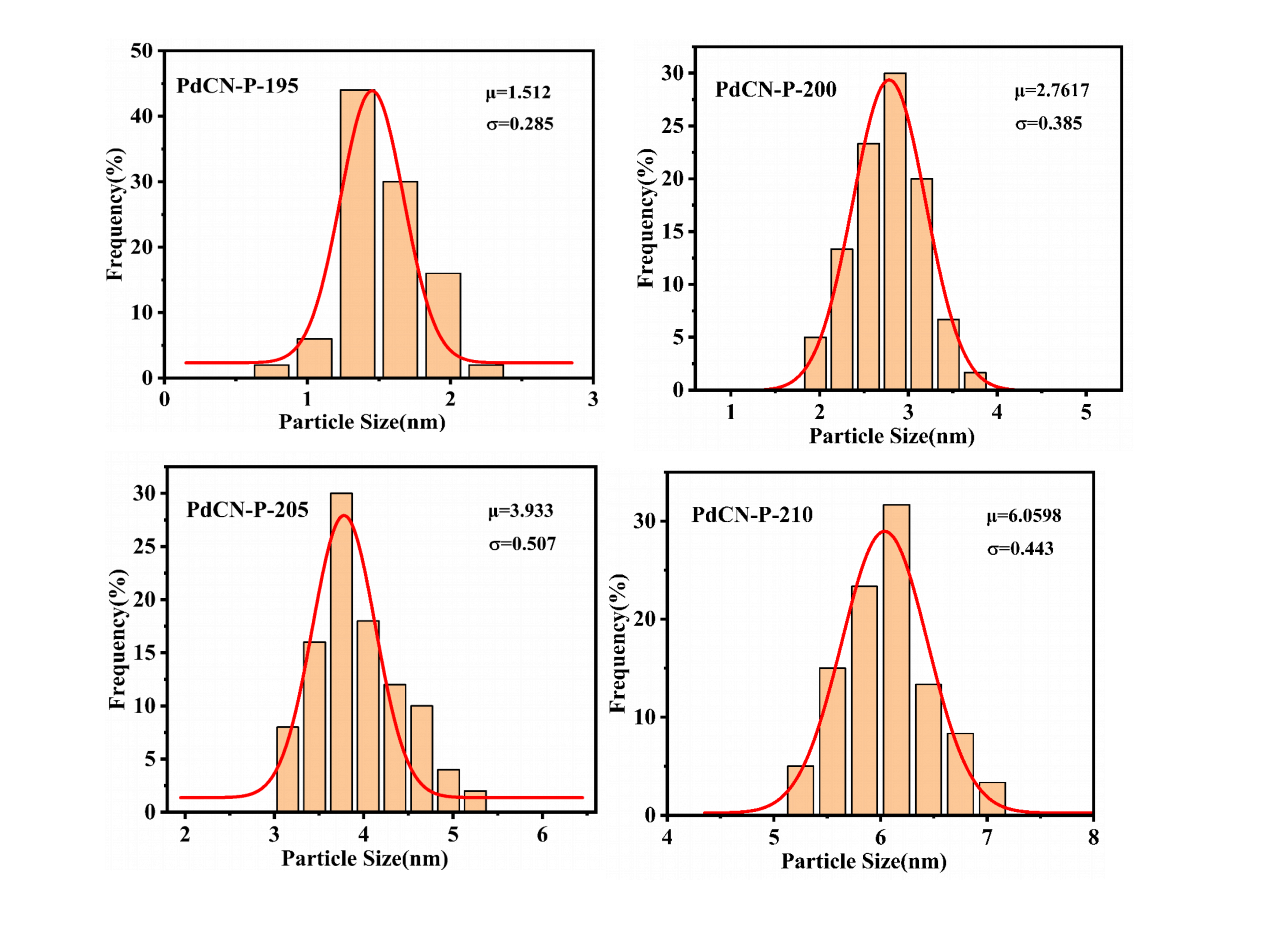


**Figure S4.** The thickness distribution of amorphous shell layers for the (a) PdCN-P-195 NCs, (b) PdCN-P-200 NCs, (c)PdCN-P-205 NCs and (d)PdCN-P-210 NCs. In these statistics, we analyzed at least 50 nanoparticles in a region on the TEM grid of each sample. μ represents the average thickness of the amorphous shell, and σ represents the standard error.


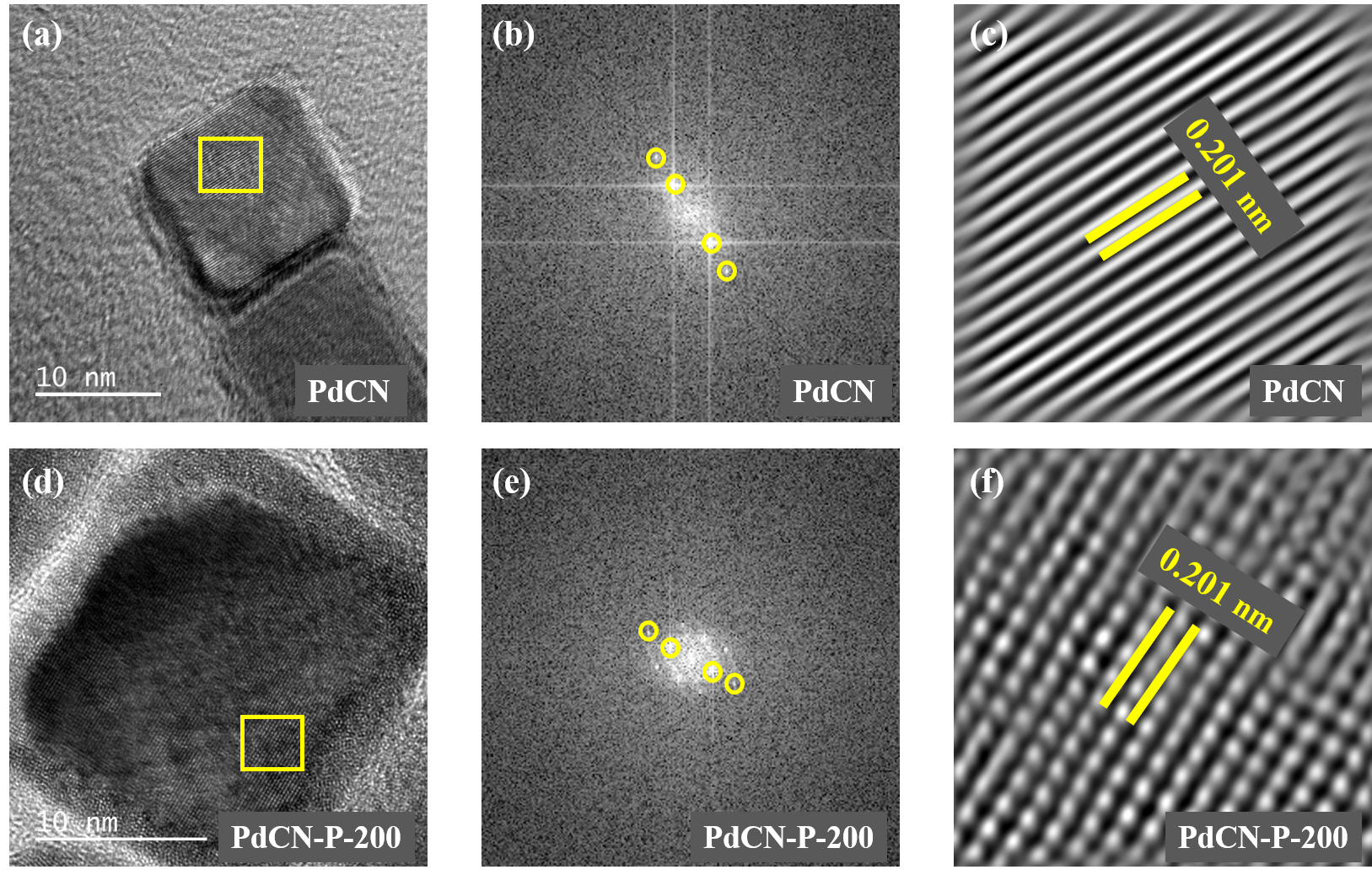


**Figure S5.** HRTEM images of (a) PdCN;(b) FFT transformation diagram of the selected area within the yellow box in Figure S5a;(c) Inverse FFT Transform of S5b. HRTEM images of (d)PdCN-P-200 (e)FFT transformation diagram of the selected area within the yellow box in Figure S5d;(f) Inverse FFT Transform of S5e.

In this study, Pd-based nanoparticles with an approximate size distribution of 20 nm were synthesized. However, due to the limited penetration depth of the electron beam for nanoparticles of this size, the TEM images exhibited significant background noise. To address this, we applied inverse FFT denoising techniques on the obtained TEM images and provided corresponding TEM images of PdCN for comparison, facilitating a clearer observation of the core lattice structure in the PdCN-P-200 sample. As shown in Figure S5, the core of the PdCN-P-200 sample, after denoising via inverse FFT, exhibits characteristic features of a cubic crystal system with an interplanar distance of 0.201 nm. This distance aligns with that of the PdCN sample, confirming the structural match between the PdCN and the core of PdCN-P-200 sample.


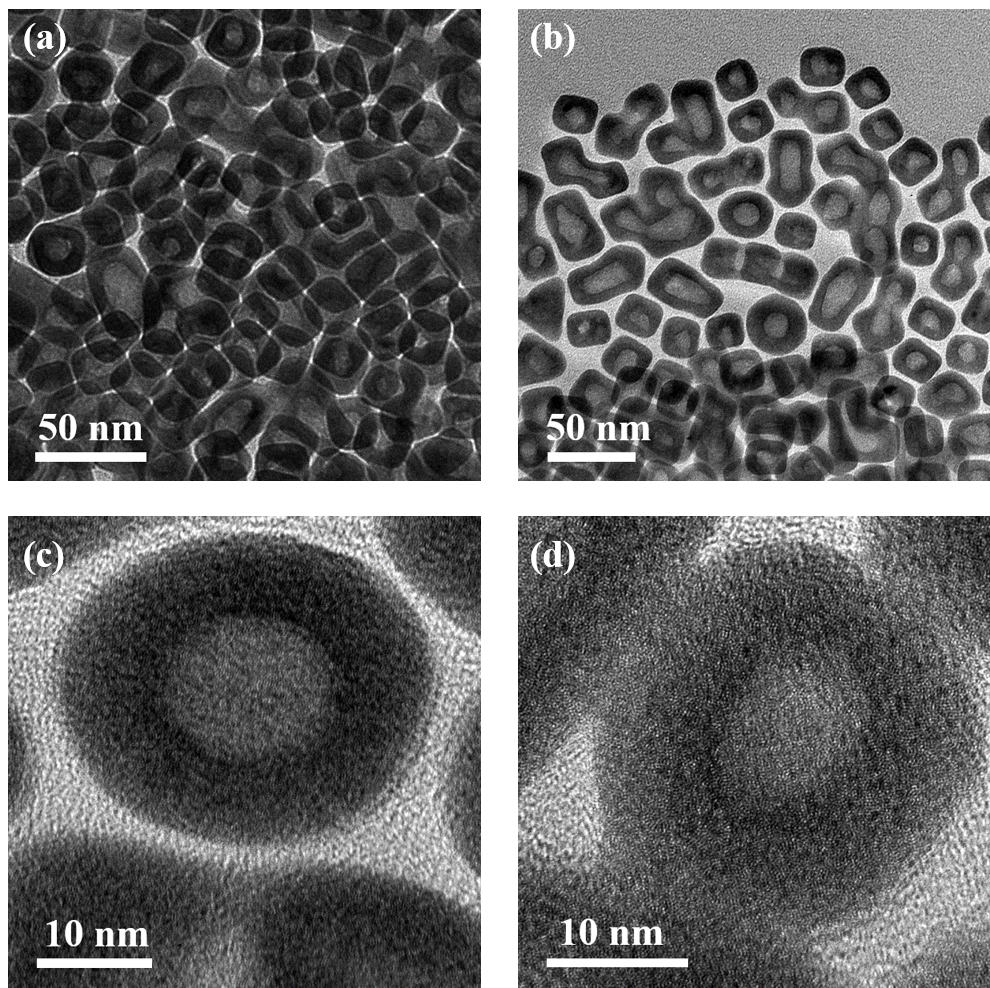


**Figure S6.**TEM and HRTEM images of (a, c) the PdCN-P-210 NCs and (b, d) the PdCN-P-220 NCs.


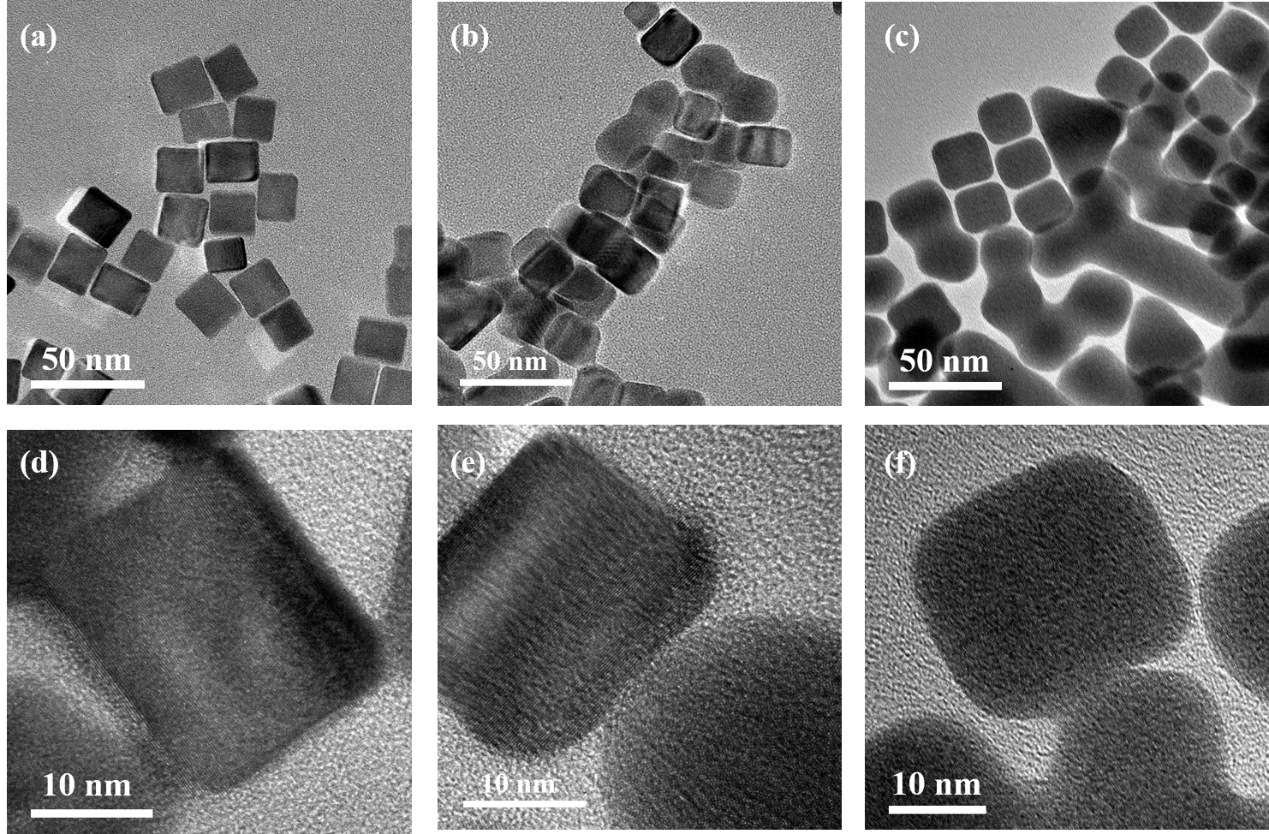


**Figure S7.** TEM and HRTEM images of (a, d)the Pd NCs, the nanoparticles obtained by reaction of Pd nanocubes with TOP at (b, e)195°C and (c, f) 200°C.

**
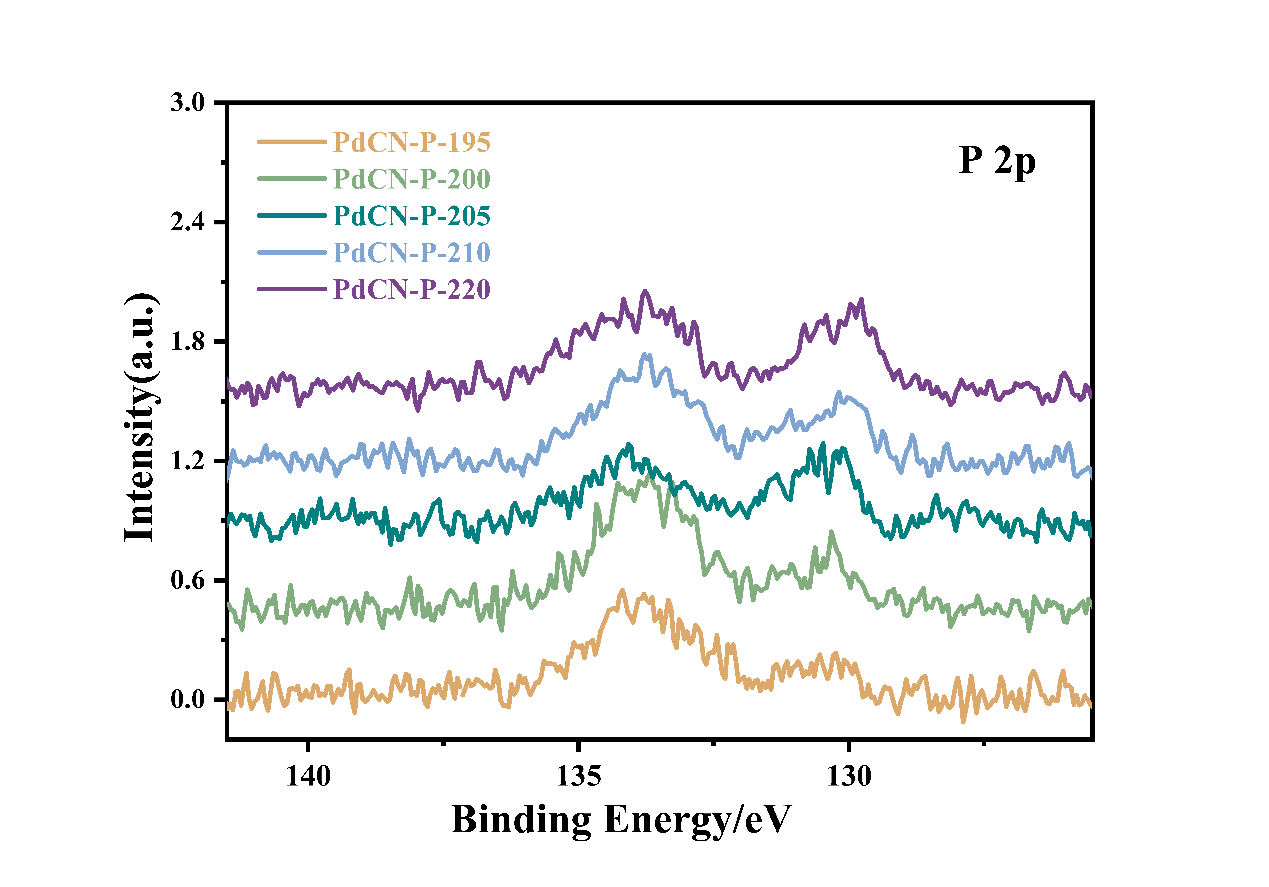
**

**Figure S8.** The high-resolution XPS spectra of P 2p for the PdCN-P-195 NCs, PdCN-P-200 NCs, PdCN-P-205 NCs, PdCN-P-210 NCs and PdCN-P-220 NCs.

**
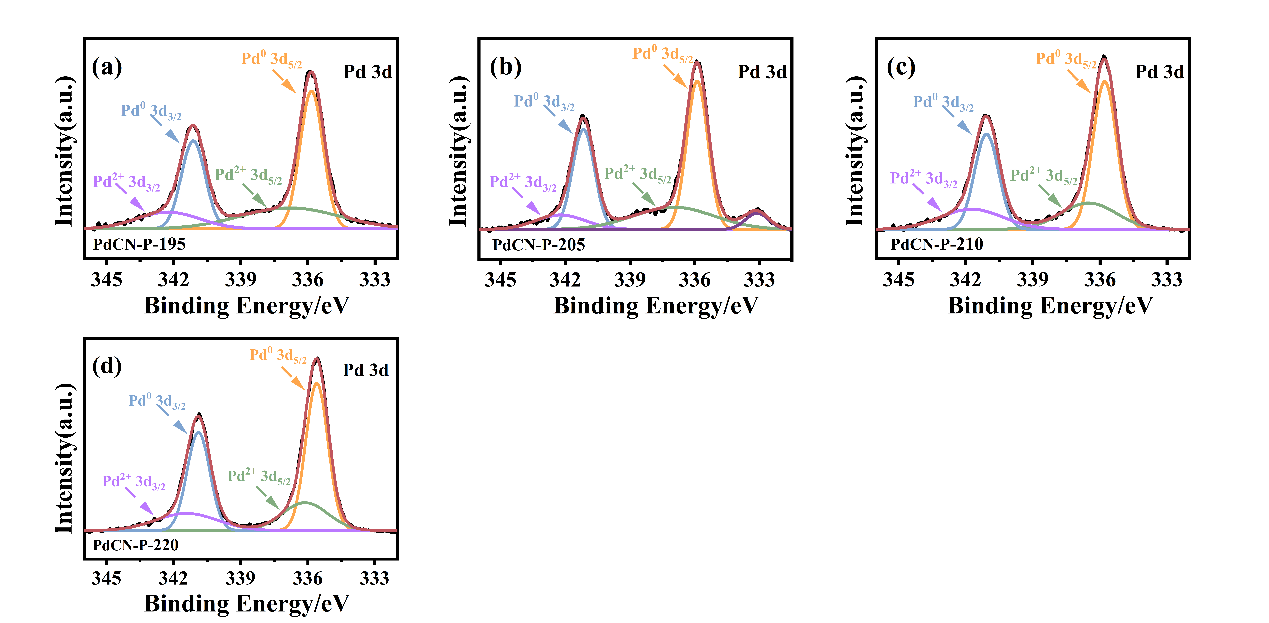
**

**Figure S9.** The high-resolution XPS spectra of Pd 3d for the PdCN-P-195 NCs, PdCN-P-205 NCs, PdCN-P-210 NCs and PdCN-P-220 NCs.

In this study, we controlled the phosphorization rate of PdCN nanocrystals by varying the reaction temperature, thereby obtaining PdCN-P nanoparticles with amorphous shells of different thicknesses. The variation in phosphorization degree and shell thickness not only influenced the spatial configuration of surface catalytic sites but also modulated the electron transfer between the crystalline core and the amorphous shell. These changes ultimately altered the electronic structure surrounding the Pd atoms in the PdCN-P samples.


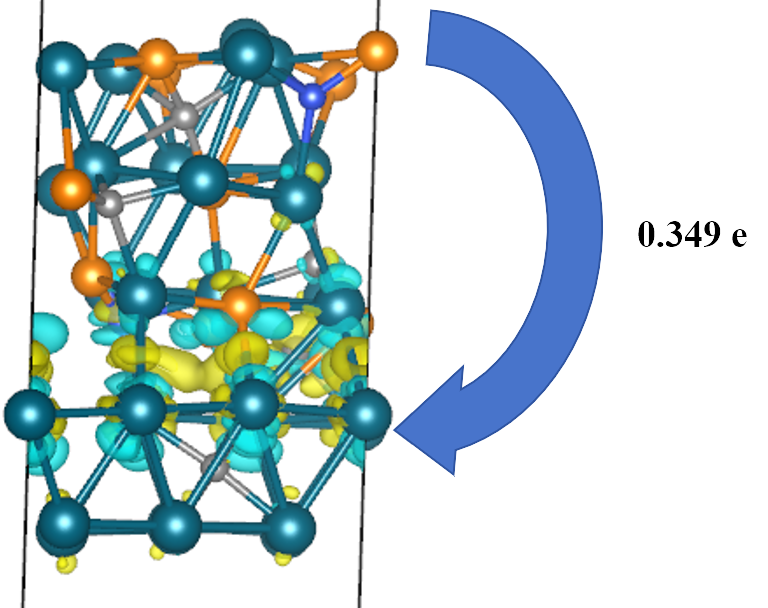


**Figure S10.** Theoretical calculation results of core-shell electron transfer.


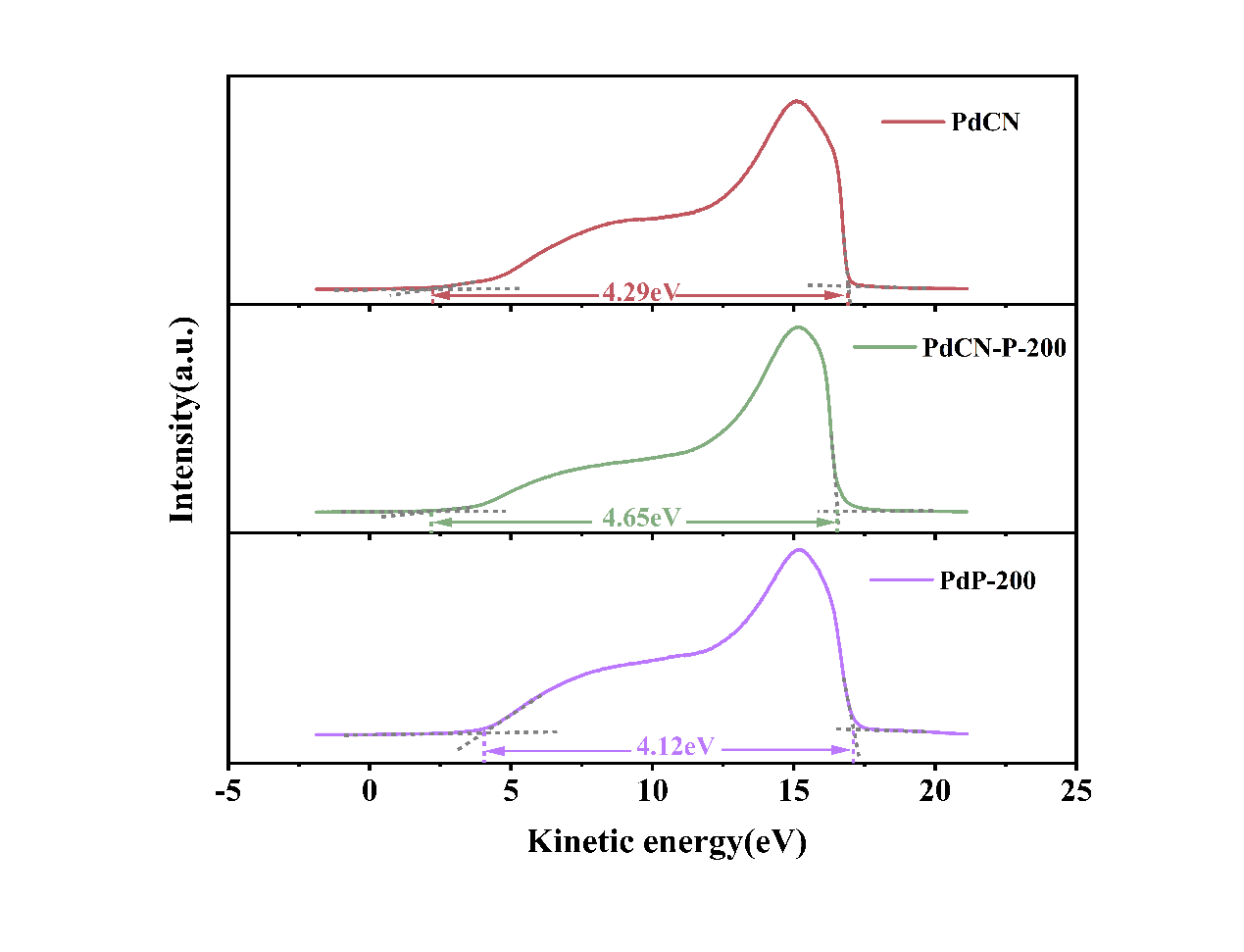


**Figure S11.** UPS spectra and work function of the PdCN NCs, PdCN-P-200 NCs and PdP NCs.


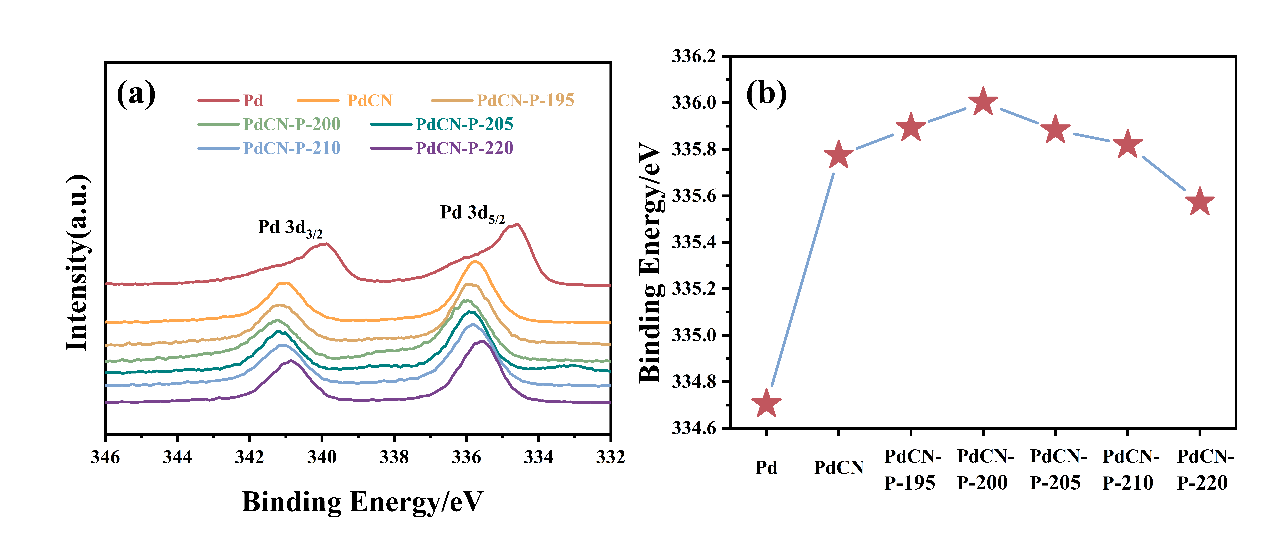


**Figure S12.** Comparison of the XPS spectra of (a) Pd 3d and (b) Pd 3d_5/2_ peak position for the Pd NCs, PdCN NCs, PdCN-P-195 NCs, PdCN-P-200 NCs, PdCN-P-205 NCs, PdCN-P-210 NCs and PdCN-P-220 NCs.


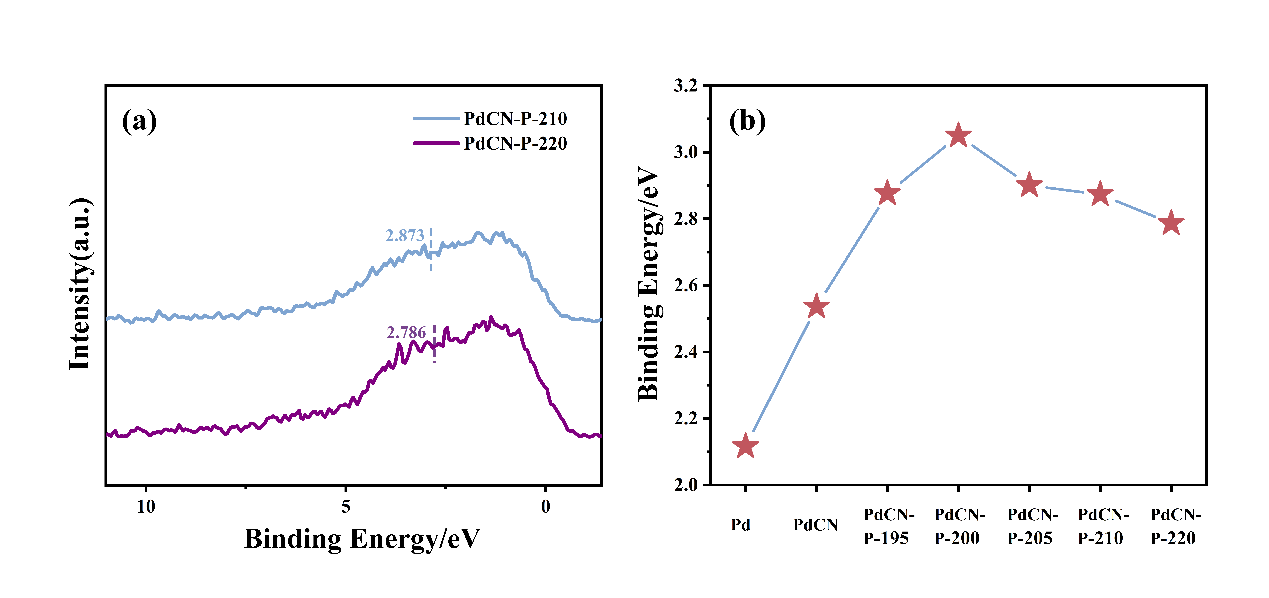


**Figure S13.** (a) XPS valence band spectra and d-band centers over the Pd 3d region for the PdCN-P-210 NCs and PdCN-P-220 NCs. (b) Comparison of the d-band centers over the Pd 3d region for the Pd NCs, PdCN NCs, PdCN-P-195 NCs, PdCN-P-200 NCs, PdCN-P-205 NCs, PdCN-P-210 NCs and PdCN-P-220 NCs.


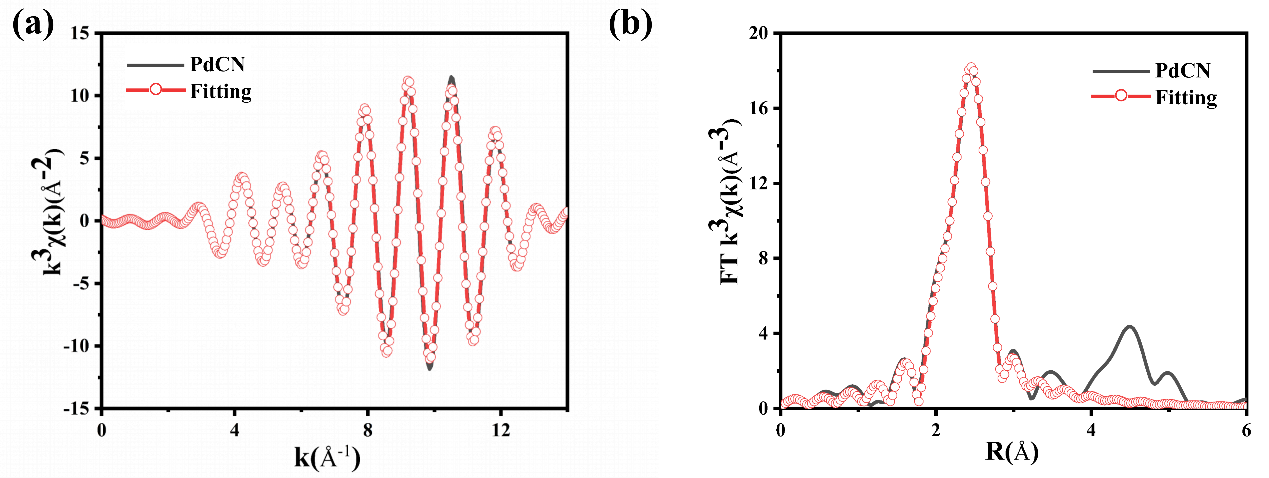


**Figure S14.** The EXAFS fitting (a) at K space spectra and (b)R space spectra of the PdCN NCs.


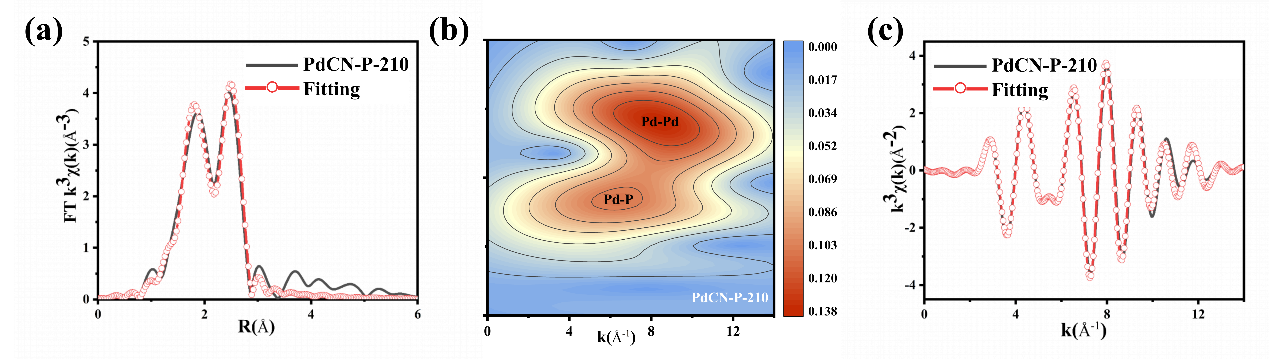


**Figure S15.** (a)The EXAFS fitting at R space spectra of the PdCN-P-210 NCs. (b) Pd K-edge wavelet transform contour plots of the PdCN-P-210 NCs. (c)The EXAFS fitting at K space spectra of the PdCN-P-210 NCs.


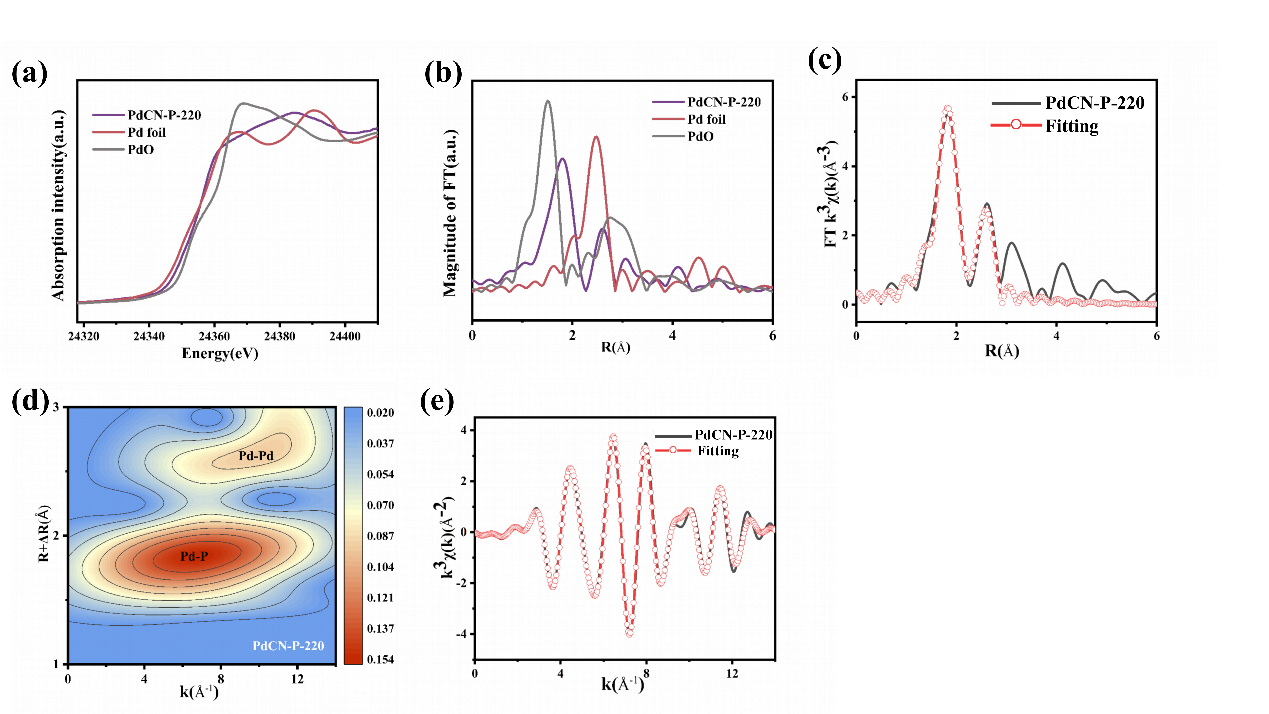


**Figure S16.** The XANES spectra (a) and the Pd K-edge Fourier transform EXAFS spectra (b) of the PdCN-P-220 NC; (c) the EXAFS fitting at R space spectra of the PdCN-P-220 NCs;(d) Pd K-edge wavelet transform contour plots of the PdCN-P-220 NCs;(e) the EXAFS fitting at K space spectra of the PdCN-P-220 NCs.


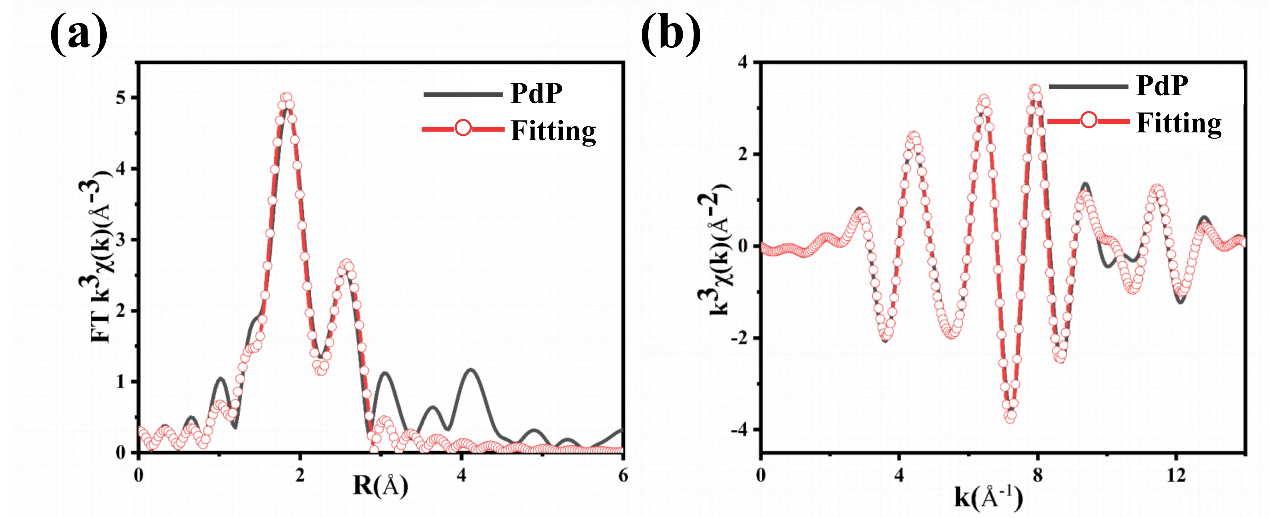


**Figure S17.** The EXAFS fitting (a) at R space spectra and (b)K space spectra of the PdP NCs.


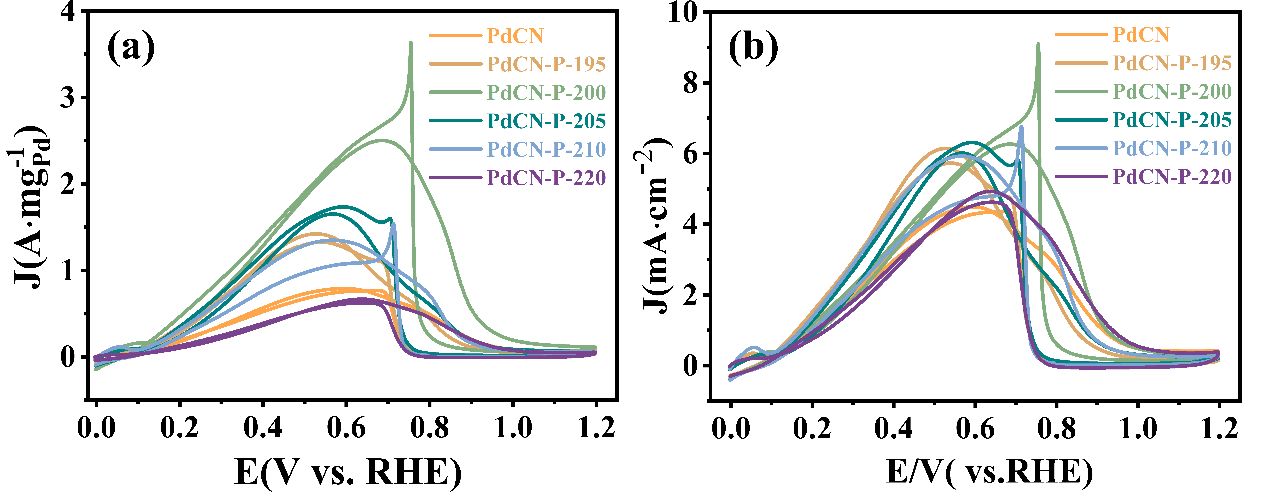


**Figure S18.** (a) Masss-normalized CV curves and (b) ECSA-normalized CV curves of the PdCN, PdCN-P-195, PdCN-P-200, PdCN-P-205, PdCN-P-210 and PdCN-P-220 catalysts in a 0.5 M H_2_SO_4_ solution containing 0.5 M HCOOH.





**Figure S19.** FAO mass activity results of PdCN-P-200 tested 10 times.





**Figure S20.** Chronoamperometric curves for 15h of PdCN-P-200 and Pd black catalysts.


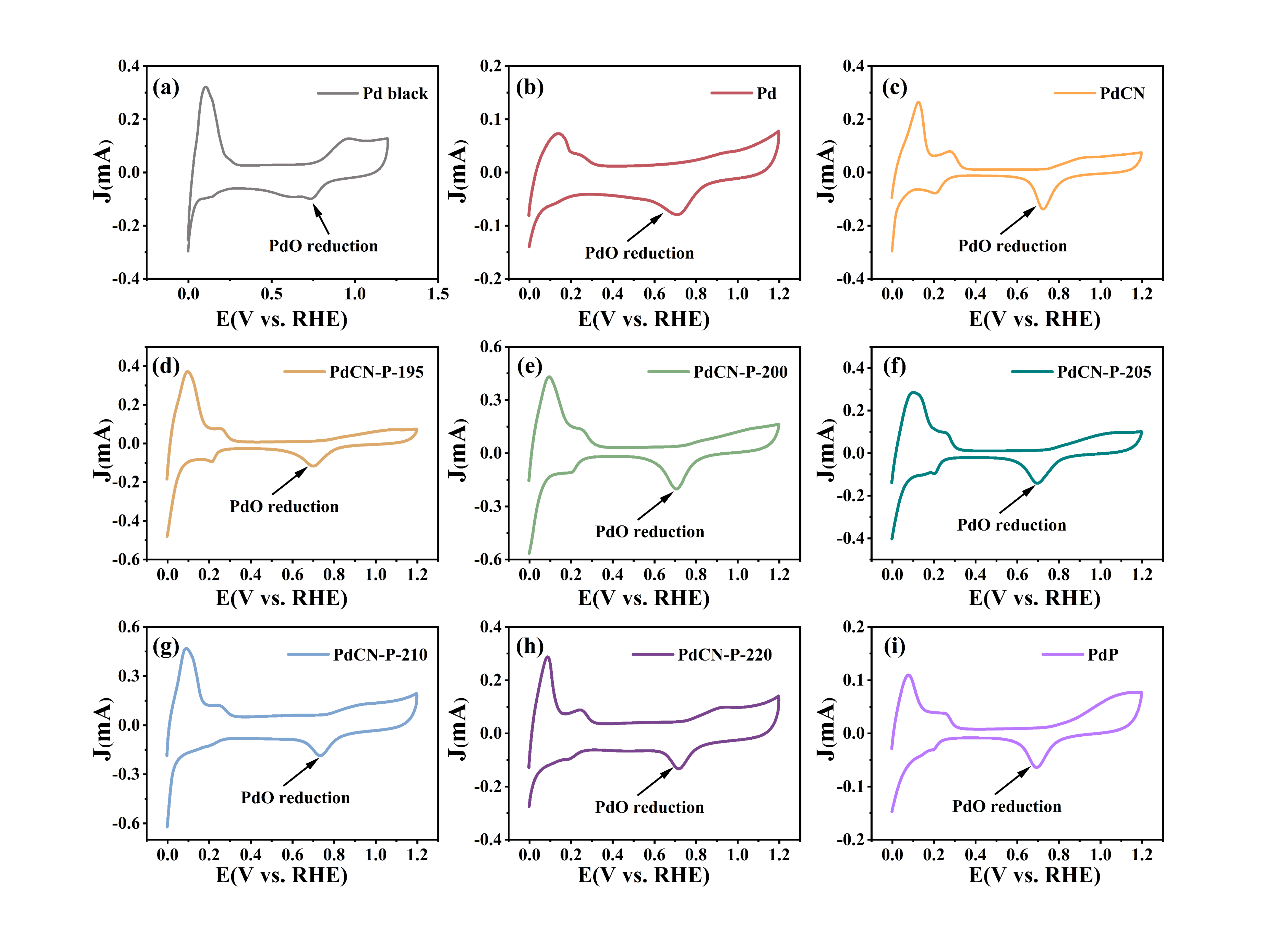


**Figure S21.** The CV curves of the all catalysts in 0.5 M H_2_SO_4_ at a scan rate of 50 mV s^−1^.


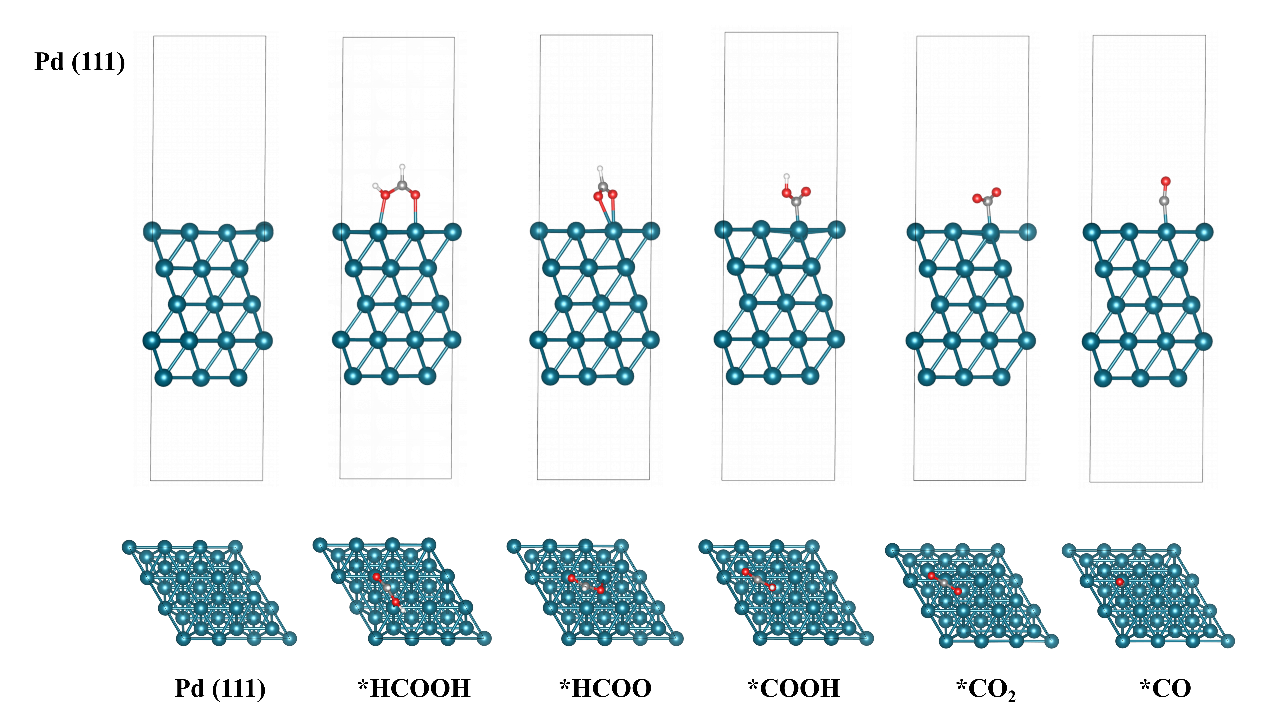


**Figure S22.** The optimized configurations of pure Pd (111) and adsorption configurations of ^*^HCOOH, ^*^HCOO, ^*^COOH, ^*^CO_2_, ^*^CO intermediates on Pd (111) surfaces. The cyan, red, grey, and white balls represent Pd, O, C, and H, respectively.


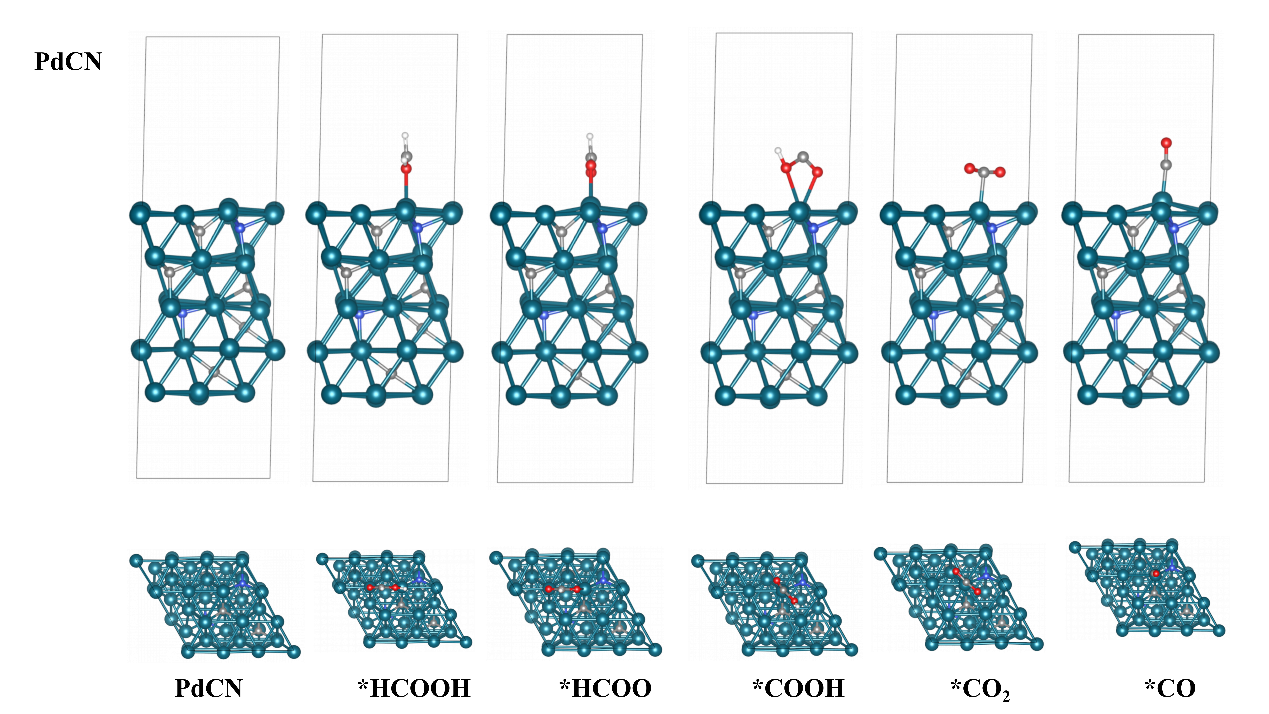


**Figure S23.** The optimized configurations of pure PdCN and adsorption configurations of ^*^HCOOH, ^*^HCOO, ^*^COOH, ^*^CO_2_, ^*^CO intermediates on PdCN surfaces. The cyan, blue, red, grey, and white balls represent Pd, N, O, C, and H, respectively.


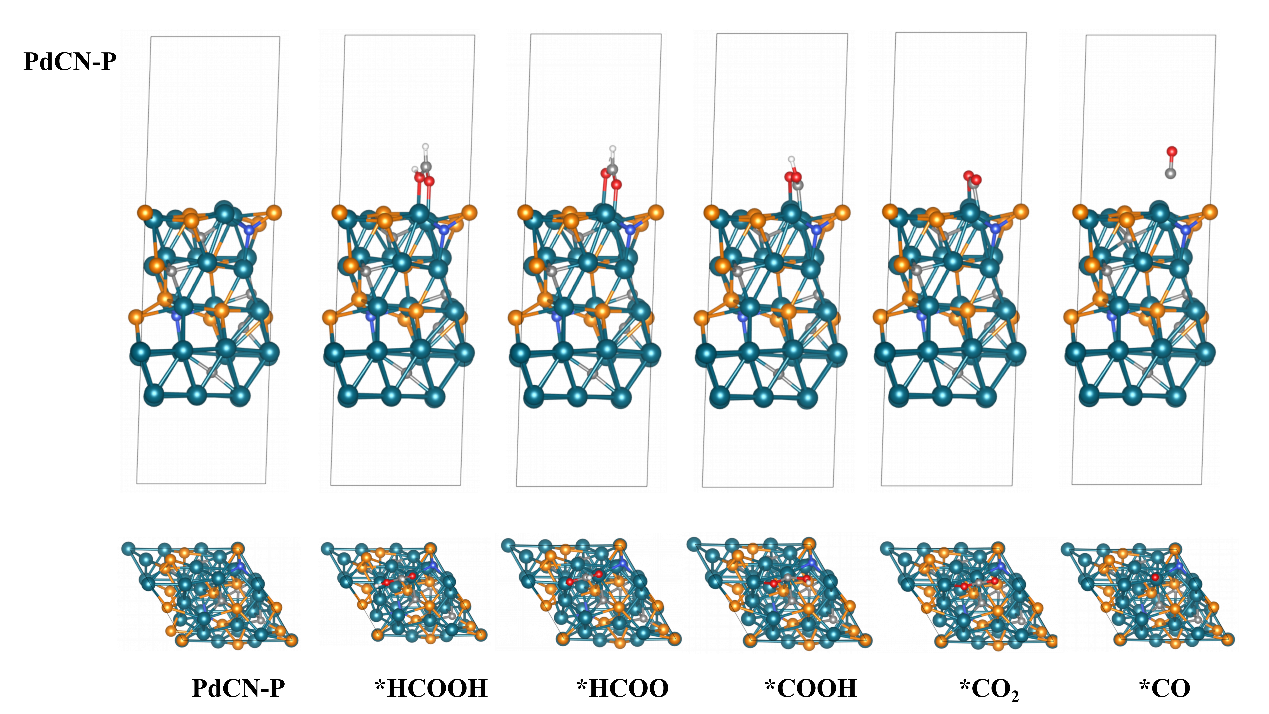


**Figure S24.** The optimized configurations of pure PdCN-P and adsorption configurations of ^*^HCOOH, ^*^HCOO, ^*^COOH, ^*^CO_2_, ^*^CO intermediates on PdCN-P surfaces. The cyan, blue, orange, red, grey, and white balls represent Pd, N, P, O, C, and H, respectively.


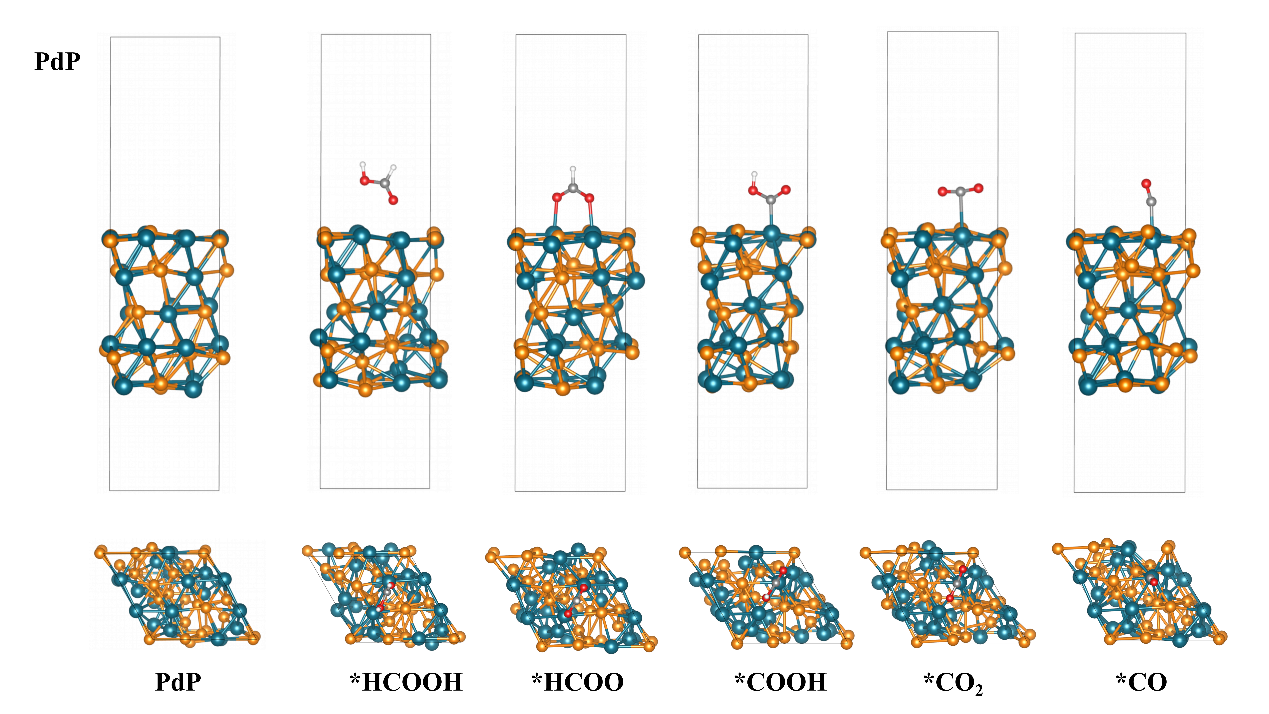


**Figure S25.** The optimized configurations of pure PdP and adsorption configurations of ^*^HCOOH, ^*^HCOO, ^*^COOH, ^*^CO_2_, ^*^CO intermediates on PdP surfaces. The cyan, orange, red, grey, and white balls represent Pd, P, O, C, and H, respectively.





**Figure S26.** Comparison of the free energy profiles of (a) Pd and (b) a-PdP catalysts via direct and indirect pathways.


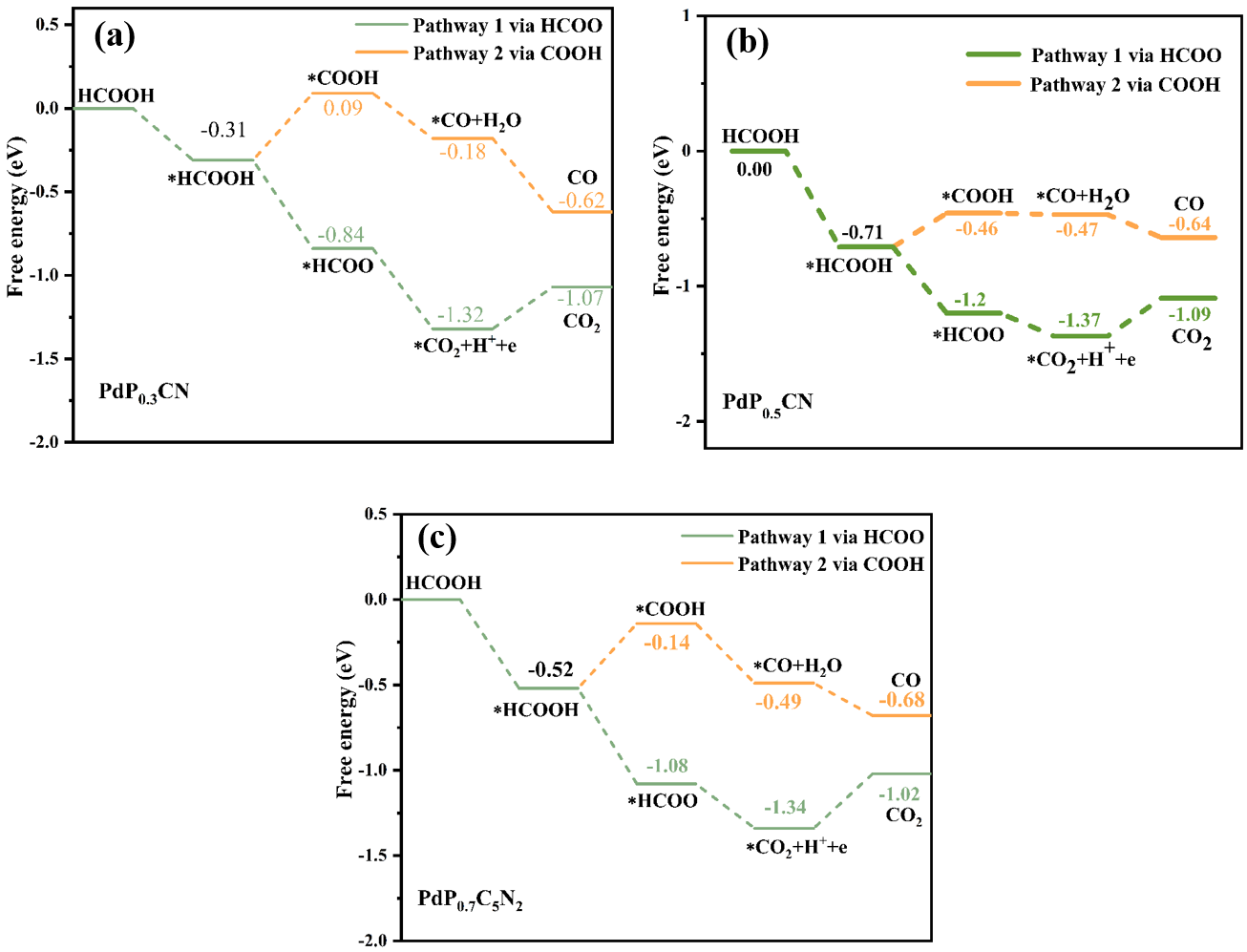


**Figure S27.** Comparison of the free energy changes of (a)PdP_0.3_CN, (b)PdP_0.5_CN and (c)PdP_0.7_CN catalysts via direct and indirect pathways.

In the original calculation section, the atomic ratio of P to Pd in the model used was 0.5. Based on this, we added the PdP_0.3_CN and PdP_0.7_CN models with the atomic ratio of P to Pd of 0.3 and 0.7, respectively. The results show that the increasing of P content influences the adsorption free energy of multiple FAO intermediates on the catalyst surface. Specifically, as the P content rises, the minimum input energy required to complete the entire oxidation process gradually increases. These values are 0.25, 0.28, and 0.32eV for PdP_0.3_CN, PdP_0.5_CN, and PdP_0.7_CN, respectively. The maximum difference in adsorption free energy between intermediates *HCOO and COOH* is observed in the PdP_0.7_CN model, indicating its better preference for the direct oxidation pathway. Additionally, intermediate *HCOOH exhibits the lowest adsorption free energy on the surface of catalyst PdP_0.5_CN, with a value of -0.71eV, suggesting that the FAO process is more easily activated on the surface of catalyst PdP_0.5_CN. These findings suggest that P doping influences the FAO performance of PdCN-P in multiple ways, resulting in a "volcanic" curve of catalytic activity as P content increases, which is consistent with our experimental observations of the specific activity of PdCN-P.


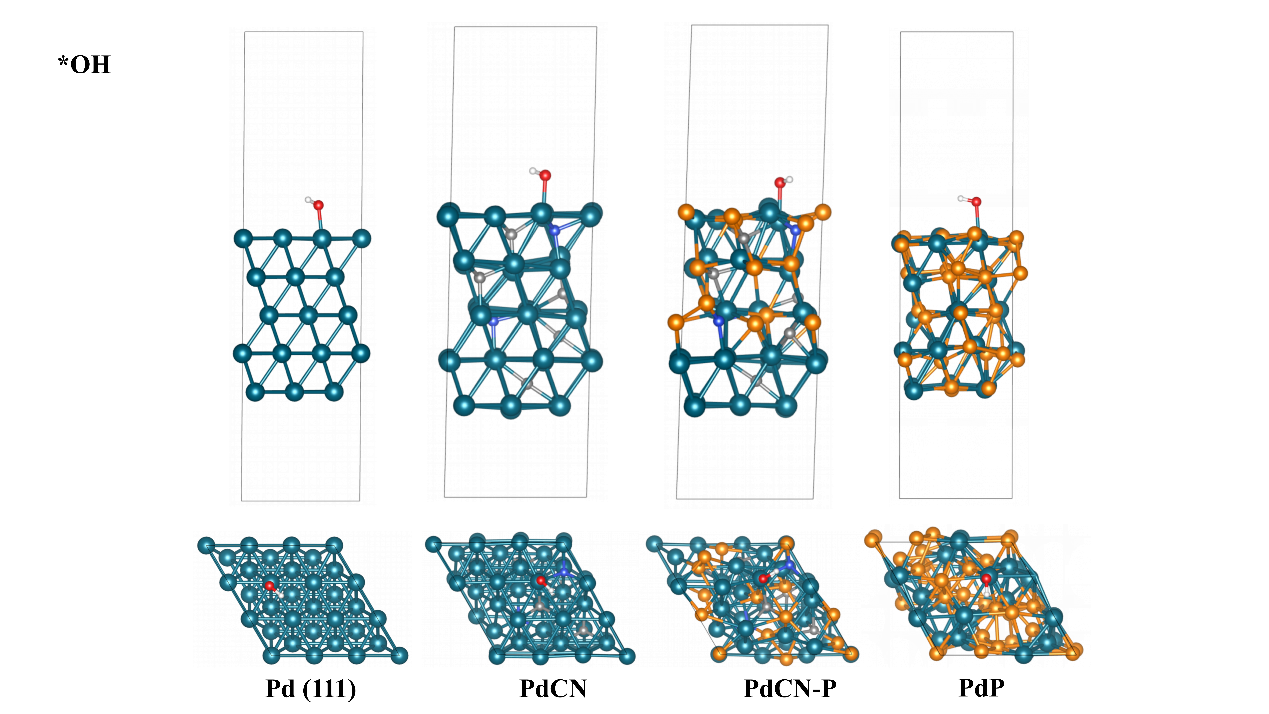


**Figure S28.** The optimized adsorption configurations of ^*^OH intermediates on Pd (111), PdCN, PdCN-P and PdP surfaces. The cyan, blue, orange, red, grey, and white balls represent Pd, N, P, O, C, and H, respectively.


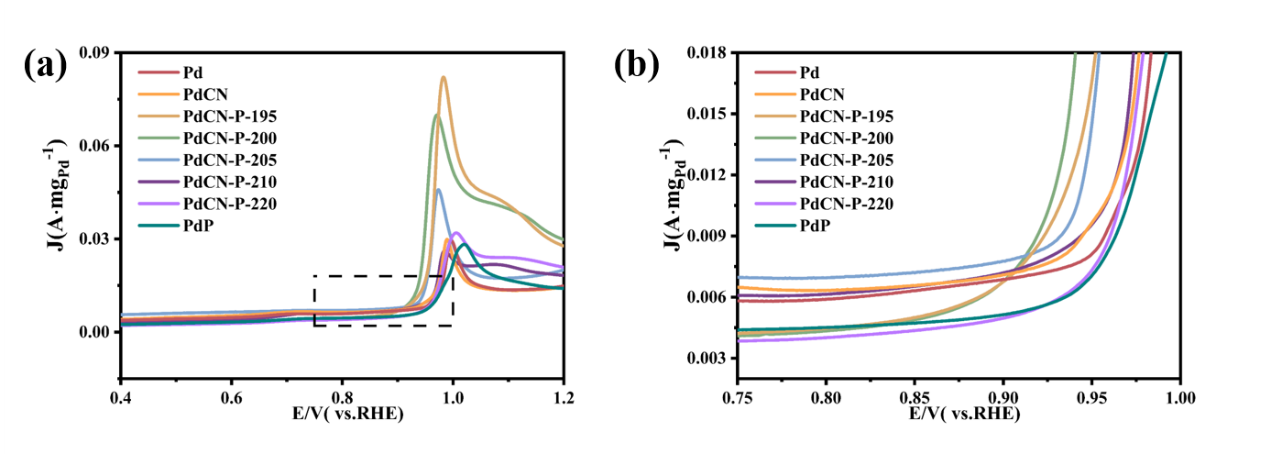


**Figure S29.** (a) CO stripping curves of the Pd, PdCN, PdCN-P-195, PdCN-P-200, PdCN-P-205, PdCN-P-210, PdCN-P-220 and PdP catalysts in 0.5 M H_2_SO_4_ solution at a scan rate of 50 mV·s^-1^and (b) the localized magnifications within the dotted box.

**3. Supplemental Tables**

**Table S1.** ICP test results of the PdCN-P-195, PdCN-P-200, and PdCN-P-205 solid samples.

| **Sample** | **Pd(wt.%)** | **P(wt.%)** |
| --- | --- | --- |
| PdCN-P-195 | 80.980 | 12.824 |
| PdCN-P -200 | 78.771 | 19.976 |
| PdCN-P -205 | 76.700 | 23.068 |

**Table S2.** Structural parameters extracted from EXAFS fitting.

| **Sample** | **Edge** | **Path** | **CN** | **R(Å)** | **σ^2^(10-^3^Å^2^)** | **ΔE_0_(eV)** | **R-factor** |
| --- | --- | --- | --- | --- | --- | --- | --- |
| Pd foil | Pd-K | Pd- Pd | 12.0 | 2.73 | 5.31 | -6.53 | 0.006 |
| PdCN | Pd-K | Pd- Pd | 9.31 | 2.73 | 5.71 | -7.52 | 0.010 |
| PdCN-P-200 | Pd-K | Pd- P | 1.91 | 2.34 | 9.69 | 0.04 | 0.015 |
|  |  | Pd- Pd | 4.42 | 2.74 | 9.98 | -5.64 |  |
| PdCN-P-210 | Pd-K | Pd- P | 2.12 | 2.33 | 6.64 | -0.56 | 0.016 |
|  |  | Pd- Pd | 4.20 | 2.75 | 9.96 | -4.30 |  |
| PdCN-P-220 | Pd-K | Pd- P | 3.01 | 2.32 | 5.36 | -0.12 | 0.010 |
|  |  | Pd- Pd | 2.73 | 2.79 | 9.94 | -3.54 |  |
| PdP | Pd-K | Pd- P | 2.61 | 2.33 | 5.19 | -0.66 | 0.016 |
|  |  | Pd- Pd | 2.80 | 2.78 | 9.99 | -3.55 |  |

Notes: CN, coordination number; R, distance between absorber and backscatter atoms. σ2, Debye-Waller factor; ΔE0, the inner potential difference between the reference compound and the experimental sample. R-factor, the goodness of fit.

**Table S3.** ICP-MS test results of the electrocatalysts.

| **Sample** | **Pd(mg/L)** |
| --- | --- |
| PdCN-P-195 | 511.3553 |
| PdCN-P -200 | 127.0507 |
| PdCN-P -205 | 300.0662 |
| PdCN-P -210 | 229.2691 |
| PdCN-P -220 | 177.5973 |

**Table S4**. Comparison of electrochemical FAO performances of Pd-based catalysts in some previous representative works and this work.

| **Catalyst** | **Electrolyte** | **Mass activity** | **Specific activity** | **Reference** |
| --- | --- | --- | --- | --- |
| PdCN-P-200 NCs | 0.5M H_2_SO_4_+0.5M HCOOH | 2.503 A∙ mg^−1^_Pd_ | 6.265 mA∙cm^-2^ | **This work** |
| B-PdRu NPAs | 0.5M H_2_SO_4_+0.5M HCOOH | 1.09 A∙ mg^−1^_Pd_ | 2.76 mA∙cm^-2^ | ^[11]^ |
| B-PdCuAu NAs | 0.5M H_2_SO_4_+0.5M HCOOH | 1.21 A∙ mg^−1^_Pd_ | 2.29 mA∙cm^-2^ | ^[12]^ |
| DP-PdCu/C | 0.5M H_2_SO_4_+0.5M HCOOH | 0.55 A∙ mg^−1^_Pd_ | 1.91 mA∙cm^-2^ | ^[13]^ |
| Pd–Pt bimetallic | 0.5M H_2_SO_4_+0.5M HCOOH | 0.77 A∙ mg^−1^ | - | ^[14]^ |
| β-PtBi_2_ | 0.5M H2SO4+0.5M HCOOH | 1.10±0.01A mg_Pt_^−1^ | - | ^[15]^ |
| Intermetallic  PdBi | 0.5M H_2_SO_4_+0.5M HCOOH | 1.35 A∙ mg^−1^_Pd_ | 4.20 mA∙cm^-2^ | ^[16]^ |
| PdAg NSs | 0.5M H_2_SO_4_+0.5M HCOOH | 0.987A∙ mg^−1^_Pd_ | 5.01 mA∙cm^-2^ | ^[17]^ |
| Pd/FeP | 0.5M H_2_SO_4_+0.5M HCOOH | 1.54 A∙ mg^−1^_Pd_ | 1.7 mA∙cm^-2^ | ^[18]^ |
| Pd6_2.5_Cu_37.5_/C | 0.5M H_2_SO_4_+0.5M HCOOH | 1.05 A∙ mg^−1^_Pd_ | - | ^[19]^ |
| PdH_0.43_-150℃ | 0.5M H_2_SO_4_+0.25M HCOOH | 1.06 A∙ mg^−1^_Pd_ | - | ^[20]^ |
| Pd3Au/C | 0.1 M HClO_4_+ 0.1 M HCOOH | 0.52 A∙ mg^−1^_Pd_ | 18.11 mA∙cm^-2^ | ^[21]^ |
| PdNiP-H  nanoparticles | 0.5M H_2_SO_4_+0.5M HCOOH | 1.454 A∙ mg^−1^_Pd_ | 1.985 mA∙cm^-2^ | ^[22]^ |
| PdBi nanodo | 0.5M H_2_SO_4_+0.5M HCOOH | 1.629 A∙ mg^−1^_Pd_ | - | ^[23]24^ |
| Pd_87_ Cu_13_ tripods | 0.5 M HClO_4_+0.5M HCOOH | 1.58 A∙ mg^−1^_Pd_ | 5.51 mA∙cm^-2^ | ^[24]^ |
| PdRuBP NAs | 0.5M H_2_SO_4_+0.5M HCOOH | 1.71 A∙ mg^−1^_Pd_ | 4.51 mA∙cm^-2^ | ^[25]^ |
| 3D-RGO/Pd-NWs | 0.5M H_2_SO_4_+0.25M HCOOH | 0.97 A∙ mg^−1^_Pd_ | - | ^[26]^ |

**4. References**

[1] M. Jin, H. Liu, H. Zhang, Z. Xie, J. Liu, Y. Xia, Synthesis of Pd nanocrystals enclosed by {100} facets and with sizes< 10 nm for application in CO oxidation. *Nano Res.* **2011**, *4*, 83-91.

[2] X. Song, L. Lü, Y. Jia, Z. Wang, Z.-A. Nan, Y.-H. Hong, D. Chen, Q. Zhang, J. Jiang, Y. Zheng, J. Xu, Z. Qiu, Q. Jiang, Y. Wang, Q. Wang, S. Dai, H. Lin, Z. Zhao, M. Chen, Z. Xie, Z.-Q. Tian, F. R. Fan, What Elements Really Intercalate into Pd Lattice When Heated in Dimethylformamide? *J. Am. Chem. Soc.* **2024**, *146*, 15320-15330.

[3] H. Tianou, W. Wang, X. Yang, Z. Cao, Q. Kuang, Z. Wang, Z. Shan, M. Jin, Y. Yin, Inflating hollow nanocrystals through a repeated Kirkendall cavitation process. *Nat. Commun.* **2017**, *8*, 1261.

[4] B. Huang, Y. Ge, A. Zhang, S. Zhu, B. Chen, G. Li, Q. Yun, Z. Huang, Z. Shi, X. Zhou, L. Li, X. Wang, G. Wang, Z. Guan, L. Zhai, Q. Luo, Z. Li, S. Lu, Y. Chen, C.-S. Lee, Y. Han, M. Shao, H. Zhang, Seeded Synthesis of Hollow PdSn Intermetallic Nanomaterials for Highly Efficient Electrocatalytic Glycerol Oxidation. *Adv. Mater.* **2023**, *35*, 2302233.

[5] a) K. Zhang, C. Wang, F. Gao, S. Guo, Y. Zhang, X. Wang, S. Hata, Y. Shiraishi, Y. Du, Recent progress in ultrafine 3D Pd-based nanocubes with multiple structures for advanced fuel cells electrocatalysis. *Coordin. Chem. Rev.* **2022**, *472*, 214775; b) H. Wang, T. Zhou, Q. Mao, S. Wang, Z. Wang, Y. Xu, X. Li, K. Deng, L. Wang, Porous PdAg alloy nanostructures with a concave surface for efficient electrocatalytic methanol oxidation. *Nanotechnology* **2021**, *32*, 355402.

[6] a) G. Kresse, J. Furthmüller, Efficiency of ab-initio total energy calculations for metals and semiconductors using a plane-wave basis set. *Computational materials science.* **1996**, *6*, 15-50; b) E. Koch, O. J. P. R. B. Gunnarsson, Density dependence of the electronic supershells in the homogeneous jellium model. *Phys. Rev. B*. **1996**, *54*, 5168; c) M. Torrent, N. A. W. Holzwarth, F. Jollet, D. Harris, N. Lepley, X. Xu, Electronic structure packages: Two implementations of the projector augmented wave (PAW) formalism. *Comput. Phys. Commun.* **2010**, *181*, 1862-1867.

[7] C. E. Calderon, J. J. Plata, C. Toher, C. Oses, O. Levy, M. Fornari, A. Natan, M. J. Mehl, G. Hart, M. Buongiorno Nardelli, S. Curtarolo, The AFLOW standard for high-throughput materials science calculations. *Comp. Mater. Sci.* **2015**, *108*, 233-238.

[8] Y. Liu, K. T. Eddie Chua, T. C. Sum, C. K. Gan, The AFLOW standard for high-throughput materials science calculations. First-principles study of the lattice dynamics of Sb_2_S_3_. *Phys. Chem. Chem. Phys.* **2014**, *16*, 345-350.

[9] J. K. Nørskov, J. Rossmeisl, A. Logadottir, L. Lindqvist, J. R. Kitchin, T. Bligaard, H. Jónsson, Origin of the overpotential for oxygen reduction at a fuel-cell cathode. *J. Phys. Chem. B.* **2004**, *108*, 17886-17892.

[10] B. Hammer, J. K. Nørskov, Theoretical surface science and catalysis—calculations and concepts. *Adv. Catal.* **2000**, *45*, 71-129.

[11] S. Liu, Z. Wang, H. Zhang, S. Yin, Y. Xu, X. Li, L. Wang, H. Wang, B-Doped PdRu nanopillar assemblies for enhanced formic acid oxidation electrocatalysis. *Nanoscale* **2020**, *12*, 19159-19164.

[12] H. Wang, X. Qian, S. Liu, S. Yin, Y. Xu, X. Li, Z. Wang, L. Wang, Boron-Doped PdCuAu Nanospine Assembly as an Efficient Electrocatalyst toward Formic Acid Oxidation. *Chem. Eur. J.* **2020**, *26*, 2493-2498.

[13] J. Geng, Z. Zhu, Y. Ni, H. Li, F. Cheng, F. Li, J. Chen, Biaxial strained dual-phase palladium-copper bimetal boosts formic acid electrooxidation. *Nano Res.* **2022**, *15*, 280-284.

[14] J. Liu, F. Li, C. Zhong, W. Hu, Clean Electrochemical Synthesis of Pd–Pt Bimetallic Dendrites with High Electrocatalytic Performance for the Oxidation of Formic Acid. *Materials* **2022**, *15*, 1554.

[15] X. Fu, H. Li, A. Xu, F. Xia, L. Zhang, J. Zhang, D. Ma, J. Wu, Q. Yue, X. Yang, Y. Kang, Phase Engineering of Intermetallic PtBi_2_ Nanoplates for Formic Acid Electrochemical Oxidation. *Nano Letters* **2023**, *23*, 5467-5474.

[16] T. Shen, S. Chen, R. Zeng, M. Gong, T. Zhao, Y. Lu, X. Liu, D. Xiao, Y. Yang, J. Hu, D. Wang, H. L. Xin, H. D. Abruña, Tailoring the antipoisoning performance of Pd for formic acid electrooxidation via an ordered PdBi intermetallic. *ACS Catal.* **2020**, *10*, 9977-9985.

[17] Z. Teng, M. Li, Z. Li, Z. Liu, G. Fu, Y. Tang, Facile synthesis of channel-rich ultrathin palladium-silver nanosheets for highly efficient formic acid electrooxidation. *Mater. Today Energy.* **2021**, *19*, 100596.

[18] Y. Bao, H. Liu, Z. Liu, F. Wang, L. Feng, Pd/FeP catalyst engineering via thermal annealing for improved formic acid electrochemical oxidation. *Appl. Catal. B: Environ.* **2020**, *274*, 119106.

[19] J. Zheng, H. Zeng, C. Tan, T. Zhang, B. Zhao, W. Guo, H. Wang, Y. Sun, L. Jiang, Coral-like PdCu Alloy Nanoparticles Act as Stable Electrocatalysts for Highly Efficient Formic Acid Oxidation. *ACS Sustain Chem Eng.* **2019**, *7*, 15354-15360.

[20] J. Zhang, M. Chen, H. Li, Y. Li, J. Ye, Z. Cao, M. Fang, Q. Kuang, J. Zheng, Z. Xie, Stable palladium hydride as a superior anode electrocatalyst for direct formic acid fuel cells. *Nano Energy* **2018**, *44*, 127-134.

[21] S.-Y. Lee, N. Jung, J. Cho, H.-Y. Park, J. Ryu, I. Jang, H.-J. Kim, E. Cho, Y.-H. Park, H. C. Ham, J. H. Jang, S. J. Yoo, Surface-rearranged Pd3Au/C nanocatalysts by using CO-induced segregation for formic acid oxidation reactions. *ACS Catal.* **2014**, *4*, 2402-2408.

[22] H. Cheng, J. Zhou, H. Xie, S. Zhang, J. Zhang, S. Sun, P. Luo, M. Lin, S. Wang, Z. Pan, J. Wang, X. J. Loh, Z. Liu, Hydrogen intercalation‐induced crystallization of ternary PdNiP alloy nanoparticles for direct formic acid fuel cells. *Adv. Energy Mater.* **2023**, *13*, 2203893.

[23] H. Xu, K. Zhang, B. Yan, J. Wang, C. Wang, S. Li, Z. Gu, Y. Du, P. Yang, Ultra-uniform PdBi nanodots with high activity towards formic acid oxidation. *J. Power Sources.* **2017**, *356*, 27-35.

[24] L. Zhang, S.-I. Choi, J. Tao, H.-C. Peng, S. Xie, Y. Zhu, Z. Xie, Y. Xia, Pd–Cu bimetallic tripods: a mechanistic understanding of the synthesis and their enhanced electrocatalytic activity for formic acid oxidation. *Adv. Funct. Mater.* **2014**, *24*, 7520-7529.

[25] Y. Xu, S. Yu, T. Ren, C. Li, S. Yin, Z. Wang, X. Li, L. Wang, H. Wang, A quaternary metal–metalloid–nonmetal electrocatalyst: B, P-co-doping into PdRu nanospine assemblies boosts the electrocatalytic capability toward formic acid oxidation. *J. Mater. Chem. A.* **2020**, *8*, 2424-2429.

[26] X. Qiu, P. Wu, L. Xu, Y. Tang, J.-M. Lee, 3D graphene hollow nanospheres@ palladium‐networks as an efficient electrocatalyst for formic acid oxidation. *Adv. Mater. Interfaces*. **2015**, *2*, 1500321.
